# Changes in Food Consumption During the COVID-19 Pandemic: Analysis of Consumer Survey Data From the First Lockdown Period in Denmark, Germany, and Slovenia

**DOI:** 10.3389/fnut.2021.635859

**Published:** 2021-03-08

**Authors:** Meike Janssen, Betty P. I. Chang, Hristo Hristov, Igor Pravst, Adriano Profeta, Jeremy Millard

**Affiliations:** ^1^Consumer and Behavioural Insights Group, Copenhagen Business School, Frederiksberg, Denmark; ^2^Consumer Science Department, The European Food Information Council, Brussels, Belgium; ^3^Nutrition and Public Health Research Group, Nutrition Institute, Ljubljana, Slovenia; ^4^Deutsches Institut für Lebensmitteltechnik - German Institute of Food Technologies, Quakenbrück, Germany; ^5^International Centre, Danish Technological Institute, Aarhus, Denmark

**Keywords:** COVID-19, food choice, food consumption, behavior change, lockdown measures, food cultures, online survey

## Abstract

This paper focuses on changes in food consumption that occurred during the COVID-19 pandemic. Its objective is to map changes at individual consumer level and identify the influence of different factors related to the COVID-19 pandemic on changes in individual food consumption. We conducted a cross-sectional online survey among 2,680 residents of Denmark (DK), Germany (DE), and Slovenia (SI) using quota sampling for gender, age and regional distribution. Data on consumption frequencies before and during the pandemic were collected with a food frequency questionnaire in the spring of 2020 (during the first lockdown period) for important types of fresh food and non-perishable food. Our results showed that, depending on the type of food, 15–42% of study participants changed their consumption frequency during the pandemic, compared to before. In all the study countries, the food categories with the highest rates of change were frozen food, canned food, and cake and biscuits; among the food categories with lower rates of change were bread, alcoholic drinks, and dairy products. People across all three countries shopped less frequently during lockdown and there was an overall reduction in the consumption of fresh foods, but an increase in the consumption of food with a longer shelf life in Denmark and Germany. Interestingly though, we observed diverging trends in all food categories analyzed, with some people decreasing and others increasing their consumption frequencies, demonstrating that the pandemic had different impacts on people's lifestyles and food consumption patterns. Using the method of multinomial regression analysis, we identified factors significantly (*p* < 0.01, *p* < 0.05, *p* < 0.1) related to increases and decrease in individuals' consumption frequencies in different food categories. The factors include restrictions put in place in response to the pandemic (i.e., closure of physical workplaces, canteens, cafés and restaurants, schools, and childcare institutions), changes in households' grocery shopping frequency, individuals' perceived risk of COVID-19, income losses due to the pandemic, and socio-demographic factors. Interesting differences between the countries were detected, allowing insights into the different food cultures. Conclusions include implications for policy-makers and actors in the food supply chain on the issues of healthy diets, food system resilience, and behavior change.

## Introduction

Food is key to personal health [e.g., (1)], as well as to the health of the planet given that current patterns of food production and consumption have considerable environmental impacts (2). Conversely, disasters such as the COVID-19 pandemic can disrupt our food system (3) and change our relationship with food. For instance, in an effort to reduce the spread of infection, border and other logistic restrictions limiting the flow of goods and people increased the risk of food shortages due to impaired supply chains, including those related to labor shortages [as can be seen in the US and Europe, (4, 5)]. Furthermore, the partial or complete lockdown measures introduced at regional and national levels, such as the closure of schools, universities, workplaces, non-essential shops and restaurants, banned events, and travel and mobility restrictions, likely changed the way people accessed their food, where they ate, and how their food was prepared. Some of these measures have served as a further obstacle to the distribution of food to vulnerable populations. For example, some programmes that provide main meals for school children were not operational during confinement. Additionally, quarantine due to illness or coming into contact with infected people may have further restricted people's access to food.

A variety of COVID-19 related psychological changes might have also affected food-related behaviors. Even in areas with relatively low disease risks, people were exposed to extensive communication about the risks of COVID-19, which was likely to have caused some of them stress. Such people may try to cope through stress-related eating, in which they attempt to make themselves feel better by eating or drinking when under stress [e.g., (6, 7)]. For example, during lockdown in Italy, people increased their consumption of processed “comfort foods,” such as chocolate, chips, and snacks (8, 9), and in some cases this was due to anxiety about their eating habits during COVID-19 (10). A study from Denmark also observed a higher degree of emotional eating during the lockdown, e.g., increased consumption of pastries and alcohol (11). In Norway, it was found that consumption of high sugar food and beverages was greater for those with increased COVID-19 related worries and general psychological distress than the overall population (12).

Risk perception associated with COVID-19 may influence people's food purchase and consumption behaviors. For example, people may try to minimize the risk of being infected by increasing their use of delivery services, purchasing more packaged food, which is seen as being more hygienic (8), buying food with a longer shelf-life (and thus purchasing less fresh food), in order to limit their shopping trips, or eating more healthy food in an attempt to boost their immune system [e.g., (13)]. Additionally, people's concern about possible food shortages may have influenced purchasing behavior, e.g., stocking up on certain foods [e.g., (8)].

It has been shown recently that COVID-19 might present additional health risks due to the metabolic impact of overeating under conditions of home confinement (14). Ammar et al. (44) reported an increase in unhealthy eating patterns based on their international survey on physical activity and eating behavior (*N* = 1,047, April 2020), something that was also observed during lockdown in a Polish national cross-sectional study (*N* = 1,097) by Sidor and Rzymski (15). About half of the participants reported more eating and snacking, while these tendencies were more pronounced in overweight individuals (15).

In Italy, which was affected much earlier and more seriously by COVID-19 than most other European countries in the first wave of the virus, a total lockdown was introduced at the national level in March 2020. A study by Scarmozzino and Visioli (9) was conducted on 1,939 participants (using a snowballing sampling approach) in April 2020 and showed that about 20% of them gained weight. This study also found and highlighted the increased consumption of processed “comfort foods,” such as chocolate, desserts, and snacks. These observations were partially confirmed by a food consumption study which investigated changes in the sale of food in over 10,000 Italian stores (8), showing an increase in the consumption of pasta, flour, eggs, long-life milk and frozen foods, alongside a reduction of fresh food purchases. This study also reported a drop in the sale of snacks, particularly sweet ones, in relation to homemade desserts, although there was an increase in savory snacks. Interestingly, the results of a COVIDiet Study, conducted on a very large sample (*N* = 7,514; snowball sampling approach) in Spain (a country also severely affected by COVID-19) showed that confinement in general led to the adoption of healthier dietary behaviors, measured as adherence to the Mediterranean diet (13).

While the above-mentioned studies focused on the general population, some studies specifically targeted younger people. A study of 820 adolescents (aged 10 to 19 years) from Italy, Spain, Chile, Colombia, and Brazil showed that COVID-19 confinement notably influenced dietary habits and modified consumption of both processed foods and fruits and vegetables (16). Gallo et al. (45) investigated the impact of COVID-19 isolation measures on Australian university students and observed increased snacking frequency and the energy density of consumed snacks. Increased energy intake was observed in females (but not males), while physical activity was impacted for both sexes – the proportion of students with “sufficient” physical activity levels was about 30% lower, in comparison with data collected in the years 2018 and 2019.

Studies by consulting companies – addressing changes in shopping behavior during COVID-19 across different product categories (food and others) – reported a marked shift across all categories to “mindful” shopping, “trading-down” to less expensive items (17), and in particular a strong focus on “essentials” (17–19). Groceries was the only product category in which consumers across all countries consistently anticipated spending more (17, 19).

The above literature regarding changes in food purchase/consumption patterns during COVID-19 documents general trends, but does not relate them to specific changes in people's circumstances due to the pandemic and resulting lockdown. Making such linkages is important in order to be able to identify the mechanisms underlying such changes, so that more accurate projections of the effects of COVID-19 can be forecast, and measures can be effectively targeted toward minimizing their negative effects on food consumption. Therefore, the main aim of our research was to understand the changes in food consumption behavior and identify the factors influencing individual changes in the food consumption frequencies of different food categories, such as fresh food, preserved food, sweet snacks, and alcoholic drinks.

To do this, we examined three countries that were similarly affected by COVID-19 infection rates in the first wave, but which varied in the extent of their lockdown measures: namely, Denmark, Germany, and Slovenia. The specific examples of government measures in the three study countries (Supplementary Table 1) illustrate how different households were affected by restrictions in different ways, e.g., not everybody was required to work from home.

To avoid some confounding factors, the study was conducted simultaneously using online panel surveys in late April and early May 2020 in three European Union countries – Denmark, Germany, and Slovenia. The three countries are comparable in terms of all having prompt and extensive government restrictions imposed at the beginning of the pandemic. On the one hand, these lockdown measures considerably limited the spread of the disease at a very early stage of the first wave of the pandemic, but on the other hand, they seriously affected people's lives. Although this paper is focused on changes in food consumption, given the scale of the pandemic and its effects on the food supply system, changes in people's food-related behavior are also likely to have implications for the resilience of food systems.

## Conceptual Framework

**Figure 1:**
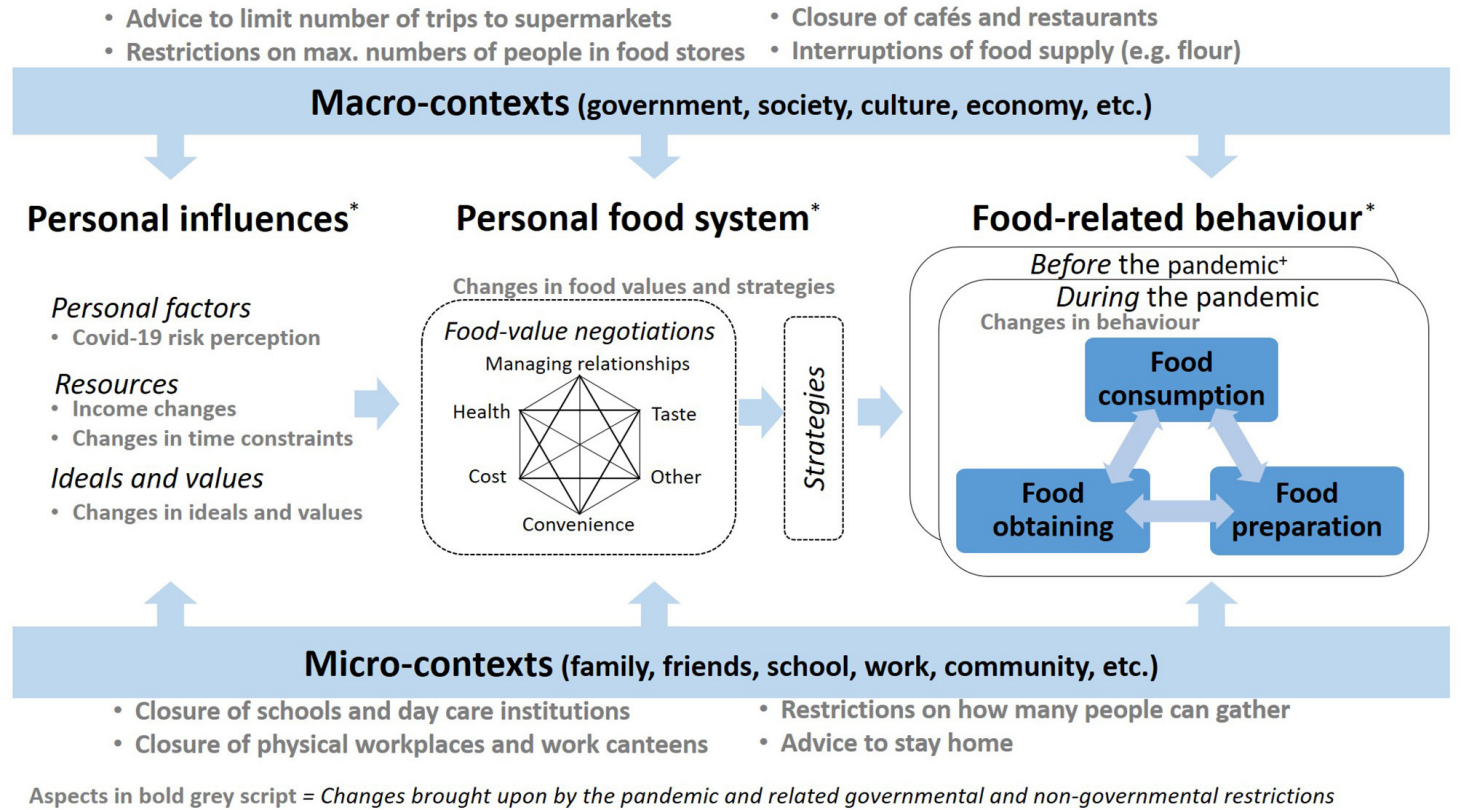
Conceptual framework of potential factors related to changes in food consumption during the pandemic [adapted from (20, 21, 23)]. *Not depicted in the figure due to space limitations: feedback loops over time between behavior, personal influences and the personal food system, as suggested by social cognitive theory [adapted from (24)]. ^+^The box on food-related behavior before the pandemic contains the same three conceptual elements as the box “during the pandemic”.

We developed a conceptual framework of factors that potentially caused changes in food consumption at the level of the individual consumer during the pandemic (Figure 1), building on two strands of literature: food choice process, and behavior change.

The interplay between food-related behaviors forms the core of our framework (Figure 1), i.e., the processes of consuming (what, where, with whom, how often), obtaining (where, how, how often), and preparing food (what, how). Food-related behaviors are influenced by the personal food system, i.e., food-related values and strategies, which in turn are influenced by personal factors, resources, and ideals (20, 21). We introduced a dynamic perspective by recognizing that food consumption *during* the pandemic is related to food consumption *before* the pandemic.

The framework further recognizes that individual-level (changes in) food consumption patterns are embedded in a complex system of multilevel factors (22), including the household level and the broader micro- and macro-context (23). We further drew upon dynamic behavior change models (24) based on Bandura's (25) social cognitive theory and concept of reciprocal determinism, postulating that personal, contextual, and behavioral factors create a feedback loop and influence each other. We thus suggest that personal experiences with changes in food-related behaviors during the pandemic potentially influence future behavior after the pandemic and might also lead to changes in personal food-related values and strategies.

Figure 1 maps the most important changes the pandemic (potentially) brought about in the study countries. This illustrates that government restrictions and lockdown measures (along with restrictions imposed by the private sector) had profound impacts on the micro- and macro-contexts of food choice. For instance, the closure of physical workplaces and the closure of schools and day care institutions interrupted people's daily life and potentially changed how, where and with whom individuals ate meals and snacks. The same applies to the closure of work canteens, cafés and restaurants, and the restrictions on private gatherings. Government recommendations to stay at home are likely to have affected how often (and where) people went food shopping.

At the personal level, we expected that the individual risk perception of COVID-19 might have caused changes in food consumption. One proposition is that people concerned about the disease would eat more healthily in order to strengthen their immune system [e.g., (13)]. An alternative proposition is that people anxious about COVID-19 might drink more alcohol and eat more comfort foods, such as snacks and cake, in order to better cope with the situation [e.g., (6, 7, 11). The pandemic also had potential impacts on households' food-related resources, i.e., money and time. Some people faced income losses, e.g., due to reduced working hours. In terms of time, households were affected by the pandemic in very different ways; some people faced severe time constraints while others had more time available for food preparation and consumption than before. In our empirical analysis, we tested the effects that pandemic-related changes at a personal level and contextual changes had on food consumption.

## Materials and Methods

### Data Collection

The online survey (compatible for both computer and hand-held devices) was conducted in the spring of 2020 (DK: April 22 to May 6; DE: April 22-27; SI: April 23-30), during the (partial) lockdown period in the selected countries. The sample contains 2,680 valid cases in total: 1,105 from Denmark, 973 from Germany, and 602 from Slovenia. Participants were recruited via consumer panel agencies with quota sampling for the age group 18+ years, gender, and region. Participants completed the online survey upon invitation. Out of 4,171 participants who had completed the survey, 1,491 were excluded (36% of initial sample) because they had not correctly responded to the two attention-check questions in the survey. The survey length, i.e., the time participants needed to complete the survey, ranged between 5 min 28 s to 38 min 56 s; the mean interview duration was 14 min 31 s.

The survey was developed in English and then translated to Danish, German and Slovenian (the complete survey can be retrieved from the Supplementary Material). The survey was pre-tested with a minimum of 10 participants in each country, including experts in food science and consumer research as well as lay people. The questionnaire contained 34 questions. To determine changes in food consumption, participants were asked to report how often they personally consumed 11 categories of fresh food, non-fresh food, convenience food, and snack food during and before the pandemic. The food frequency questionnaire contained a six-point scale ranging from *less than once a fortnight or never* to *daily*:

- fresh food (fruit & vegetables, meat, fish, dairy, bread),
- non-perishable food (frozen food and canned food),
- ready-made meals,
- sweet snacks (cake & biscuits, sweets & chocolate), and
- alcoholic drinks.

Moreover, participants reported their behavior *before* and *during* the COVID-19 pandemic in terms of:

a) from which channels (e.g., supermarkets, farm markets, home delivery) they obtained various foods (answer format: check all that apply from a list of channels),
b) the frequency of purchasing four food types: fresh vegetables and fruits, fresh fish and meat, other fresh products, and non-fresh food (answer format: six-point scale ranging from *less than once a fortnight or never* to *daily*),
c) which meals were typically prepared and consumed at home (answer format: check all that apply from a list of meals),
d) the main ways household food was prepared, e.g., from take away, from a supermarket, ready to heat/cook meals, home-made meals using either processed and raw ingredients (answer format: check all that apply from a list of food preparation ways),
e) the frequency of consuming from various eateries away from home, e.g., work canteens, cafés and restaurants, street vendors, free food in hostels (answer format: six-point scale ranging from *less than once a fortnight or never* to *daily*), and
f) whether meals in the household had been missed due to lack of food and anxiety about obtaining enough food (answer format: three-point answer scale from *never* to *frequently*).

Participants were further asked whether they had experienced certain changes due to COVID-19, including changes in household income and closure of their physical workplace.

Questions were also asked about the extent to which their household had been afflicted with COVID-19, and their own perceived risk of the disease based on three items (with a five-point answer scale from *very low* to *very high*). Finally, they reported on the demographic details of their household and themselves.

### Data Analysis

The data was analyzed at country level. The first step included paired-samples *t*-tests to detect significant differences in the mean food consumption and shopping frequencies of different food categories during the pandemic compared to before. In addition, we identified individual changes in food consumption by comparing consumption frequencies during the pandemic and before. For each of the 11 food categories, we determined whether an individual had increased, decreased or not changed their personal consumption frequency. These descriptive analyses served the aim of mapping changes in food-related behaviors during the pandemic.

The second step addressed the aim of identifying factors with a significant effect on changes in individuals' food consumption during the pandemic. We estimated multinomial logistic (MNL) regression models (maximum likelihood estimation) using STATA version 15.1 (StataCorp LLC, TX, USA). The dependent variable was the individual change in consumption frequency with the three possible outcomes “increase,” “decrease,” and “no change” in consumption frequency.

MNL regression models are designed for a nominal outcome variable with more than two levels (26). These models simultaneously estimate binary logits (i.e., the logarithm of odds of the different outcomes) for all possible outcomes, while one of the outcomes is the base category (or comparison group). In our case, the outcome “no change” served as the base category. We estimated separate models for the 11 food categories and the three countries.

The MNL regression models predict the probability *P* that a respondent increased/decreased consumption frequency subject to a set of independent variables (listed in Table 1):

(1) $$\begin{array}{r} P\left\{y_{i}=t\right\}=\frac{\exp\left(X_{it-1}^{{}^{\prime }}\;\beta_{l}\right)}{1+\;\sum_{k=1}^{J}\exp\left(X_{it-1}\beta_{k}\right)}, \end{array}$$

with *X* being a vector of independent (or predictor) variables for consumer *i, t*, and *j* representing choice alternatives from choice set *J*, and β being the parameters estimated by the model.

**Table 1:** Variables included in the multinomial logistic regression models.

**Variable**	**Definition**	**Measurement**	**Link to conceptual framework**
Yi	Change in consumption frequency	(1) “increase”	Food-related
		(0) “no change”	behavior
		(−1)“decrease”	
x1i	Changes in food shopping frequency	Continuous variable: sum scale of changes in shopping frequency in four food categories (fresh fruit & vegetables, fresh meat & fish, other fresh food, non-fresh food)	Food-related behavior
x2i	COVID-19 risk perception	Continuous variable: Sum scale of three items measured on a 5-point interval scale	Personal factor
x3i	Closure of physical workplace	(1) “not affected”	Micro-context
		(2) “affected”: respondent's physical workplace was closed during the first lockdown	
x4i	Closure of work canteens	(1) “not affected”	Micro-context
		(2) “affected”: respondent had eaten there at least once a week before the pandemic but not during the first lockdown	
x5i	Closure of cafés and restaurants	(1) “not affected”	Macro-context
		(2) “affected”: respondent had eaten there at least once a week before the pandemic but not during the first lockdown	
x6i	Income loss due to pandemic	(1) “not affected”	Personal resources
		(2) “affected”: Respondent had lost income due to the pandemic	
x7i	Household composition	(1) “household with children”	Micro-context
		(2) “single-person household”	
		(3) “household with 2+ adults, no children living in the household”	
x8i	Gender	(1) “female”	Personal factor
		(2) “male”	
x9i	Education	(1) “lower secondary or equivalent”	Personal factor
		(2) “upper secondary or equivalent”	
		(3) “university or higher degree”	
x10i	Age group	(1) “19–35 years of age”	Personal factor
		(2) “36–49 years of age”	
		(3) “50–65 years of age”	
		(4) “66+”	
x11i	Eating frequency before pandemic	Continuous variable: 6-point interval-scale	Food-related behavior

The relative probability of an “increase”/“decrease” of consumption frequency compared to the base outcome “no change” is calculated as follows:

(2) $$\begin{array}{r} \frac{\mathrm{Pr}\left(y_{\left(increase\right)}\right)}{\mathrm{Pr}\left(y_{\left(no\;change\right)}\right)}=\exp\left(X\beta^{increase}\right) \end{array}$$

(3) $$\begin{array}{r} \frac{\mathrm{Pr}\left(y_{\left(decrease\right)}\right)}{\mathrm{Pr}\left(y_{\left(no\;change\right)}\right)}=\exp\left(X\beta^{decrease}\right) \end{array}$$

The coefficients reported in the Supplementary Material are odds ratios (OR):

(4) $$\begin{array}{r} OR=\;\frac{\frac{\mathrm{Pr}\left(y=increase\;|\;x+1\right)}{\mathrm{Pr}\left(y=no\;change\;|\;x+1\right)}}{\frac{\mathrm{Pr}\left(y=increase\;|\;x\right)}{\mathrm{Pr}\left(y=no\;change\;|x\right)}} \end{array}$$

The models were estimated as “full models,” i.e., each model contained the whole set of independent variables listed in Table 1. The choice of independent variables predicting changes in food consumption frequency was guided by our conceptual framework (Figure 1). The models included food-related behaviors, personal factors and resources, and contextual factors. The latter were operationalised as respondent-specific variables: based on our questionnaire, we could determine whether a respondent was directly affected by a change in the macro- or micro contexts due to the pandemic, e.g., whether the respondent's physical workplace had been closed or whether the respondent had frequently eaten out-of-home before but not during the pandemic.

Most of the independent variables were direct measures from the questionnaire, two variables were sum scales (see Table 1). The variable “changes in food shopping frequency” is the sum scale of changes in food shopping frequency in four food categories (fresh fruit & vegetables, fresh meat & fish, other fresh food, non-fresh food), measured on a six-point frequency scale before and during the pandemic. The variable “COVID-19 risk perception” is the sum scale of three items measured on a five-point rating scale (“The likelihood of any member of your household to become infected by the virus,” “The likely severity of the virus for any member of your household,” “The level of your anxiety concerning the potential impact of the virus on your household”) adapted from Kwok et al. (46). The scale was tested for reliability and displayed good Cronbach's alpha values of 0.77 (DK), 0.82 (DE), and 0.74 (SI).

## Results

The results chapter starts with a description of the socio-demographic composition of the sample (section Socio-demographic characteristics of the sample) and the main COVID-19 impacts (section Main COVID-19 impacts), before presenting the observed changes in food-related behaviors (section Changes in food-related behaviors), and the analysis of factors significantly related to increases and decreases of food consumption frequencies (section Factors related to changes in food consumption frequencies).

### Socio-Demographic Characteristics of the Sample

In terms of gender, the samples in all three countries are close to the distribution in the national populations, i.e., ~50–50 (Table 2). The age distribution in the samples is also generally reflective of the national population, with the following observations:

- The 19–49 age groups in Denmark are a little under-represented, and in Slovenia somewhat over-represented.
- The 50–65 age group is somewhat over-represented in all three countries.
- The 66+ age group is somewhat over-represented in Denmark and under-represented in both Germany and Slovenia.

**Table 2:** Socio-demographic composition of the sample.

		**Denmark (*N* = 1,105)**	**Germany (*N* = 973)**	**Slovenia (*N* = 602)**
			**Sample %**	
Gender	Female	53.3	57.3	50.2
	Male	46.6	42.5	49.8
	Other	0.2	0.1	0.0
Age	*Mean age in years (SD)*	*54.9 (14.1)*	*48.9 (16.0)*	*44.1 (13.5)*
	19–35 years of age	14.3	23.0	29.7
	36–49 years of age	12.2	25.3	33.6
	50–65 years of age	46.6	34.7	30.9
	66+	26.9	17.0	5.8
Education	Lower secondary or equivalent	21.5	10.5	4.0
	Upper secondary or equivalent	45.9	54.1	62.4
	University or higher degree	32.6	35.5	33.4
Household composition	Households with children	17.3	23.3	41.0
	Single-person households	27.0	29.4	8.8
	Households with 2+ adults without children living in the household	55.7	47.3	50.2

Denmark's sample of educational level is very similar to the country average, whilst in Germany and Slovenia the sample is somewhat skewed toward tertiary education and in Slovenia the lower secondary group is under-represented.

The household composition in the sample also slightly deviates from the population. In Denmark's sample, households with children are somewhat under-represented and households with two or more adults are over-represented. In Slovenia's sample, households with children are over-represented and single-person households are under-represented.

### Main COVID-19 Impacts

Table 3 presents important changes brought by the pandemic on the sample population, where relevant compared with national and EU28 data. When related to the changes in food-related behavior reported by respondents discussed below, this enables international comparisons to be made with potentially important lessons for food behavior and culture, food systems, food policy, and crisis management.

**Table 3:** Changes brought by the COVID-19 pandemic.

	**Denmark (*N* = 1,105)**	**Germany (*N* = 973)**	**Slovenia (*N* = 602)**
***A. COVID-19 impacts on sample households***	**Sample %**	**Sample %**	**Sample %**
Infected members	6.5	2.6	3.8
Isolation or quarantine	6.1	2.6	6.2
Hospitalization	0.2	0.3	0.2
***B. National COVID-19 impacts until end April 2020^a^***			
Cumulative cases/100,000	*155.5^+^*	*189.9^+^*	*68.2^+^*
Cumulative deaths/100,000	*7.6^+^*	*7.5^+^*	*4.3^+^*
***C. Risk perception of COVID-19 reported by sample households (5-point scale from “1=very low” to “5=very high”)***	**Mean**	**Mean**	**Mean**
1. The likelihood of any member of your household to become infected by the virus.	2.4	2.6	2.2
2. The likely severity of the virus for any member of your household.	2.6	2.7	2.6
3. The level of your anxiety concerning the potential impact of the virus on your household.	2.5	2.8	2.7
***D. Changes in micro- and macro-context of sample households***	**Sample %**	**Sample %**	**Sample %**
Closure of your (physical) workplace	43.3	29.2	73.6
Eating at work canteens before but not during the pandemic^c^	17.1	14.8	17.2
Eating at cafés or restaurants before but not during the pandemic^c^	7.8	22.0	22.8
***E. Income change during COVID-19 reported by sample households***	**Sample %**	**Sample %**	**Sample %**
Income loss	9.1	23.4	53.2
Income gain	1.4	2.7	1.0
***F. Food poverty & anxiety reported by sample households (measured on 3-point scale “never,” “occasionally,” “frequently”; sample % refer to proportion who answered “occasionally” and “frequently”)***	**Sample %**	**Sample %**	**Sample %**
Missed meals before pandemic	9.9	6.4	11.8
Missed meals during pandemic	10.2	5.2	12.8
Anxiety about acquiring food before pandemic	11.2	2.9	7.8
Anxiety about acquiring food during pandemic	17.6	17.8	31.6
***National poverty data^b^***			
At risk of poverty (national data)	*16^+^*	*17^+^*	*14^+^*

a *The respective EU28 values (per 100,000) are 206.8 cumulative cases and 23.9 cumulative deaths (47)*.

b *The EU28 value (for 2019) is 22% (43)*.

c *These percentages refer to the share of participants who had indicated in the questionnaire that they had eaten at work canteens and cafés/restaurants, respectively, before but not during the pandemic*.

+ *This data refers to national data and not to our sample data*.

#### COVID-19 Impacts and Risk Perception

In terms of nationally reported COVID-19 cases and deaths, all three countries do much better than the EU28 average up until the end of April 2020, and all three have a lower urbanization rate than EU28 (although Germany is only just below). One explanation for this is the evidence that cities constitute the epicenter of the pandemic, particularly because of their high levels of connectivity and air pollution, both of which are strongly correlated with COVID-19 infection rates, although there is no evidence to suggest that density *per se* correlates to higher virus transmission (27). This is loosely shown in our three countries, but other factors clearly also play important roles, probably including policy and regulatory interventions, as well as culture and attitudes to behavior more generally.

In terms of COVID-19 impacts on the sample households, the questionnaire contained three separate questions asking whether any household member had been (a) infected with COVID-19 or had symptoms consistent with COVID-19, (b) in isolation or quarantine because of COVID-19, and (c) in hospital because of COVID-19. Denmark's sample experienced significantly more infected household members and household members in isolation/quarantine than Germany (*Z*-tests for comparison of proportions, *p* < 0.001). The number of infected household members in Slovenia was higher than in Germany and lower than in Denmark but the differences were not significant. Slovenia's sample also experienced significantly more household members in isolation/quarantine than Germany (*Z*-tests for comparison of proportions, *p* < 0.01). All three countries had relatively low hospitalization rates. The sample data tend to align with the three countries' overall population case and death rates and with urbanization rates. Interestingly, not all participants who indicated that a household member had been infected with COVID-19 or had symptoms consistent with COVID-19 also reported that a household member had been in isolation or quarantine. A possible explanation is that in the early phase of the pandemic in the study countries (i.e., until the beginning of March), people with symptoms consistent with COVID-19 were often not tested for COVID-19 and not necessarily asked to self-isolate or go into quarantine.

COVID-19 risk perception in the sample households was, on average, low to medium in the overall sample (Table 3, topic C.), with some statistically significant differences between the countries (comparison of mean values with ANOVA). Regarding the likely severity of the virus for any member of the household (item 2), we observed no significant differences between the countries. Regarding the likelihood of any member of the household to become infected by the virus (item 1), a significantly (*p* < 0.001) higher mean value was observed in Germany, followed by Denmark and Slovenia, perhaps reflecting the higher overall COVID-19 impact in Germany and Denmark, and the relatively strict lockdown measures in Slovenia. At the same time, the level of anxiety concerning the potential impact of the virus on the household (item 3) was significantly (*p* < 0.01) higher in Slovenia and Germany compared to Denmark, perhaps because of the closer geographical proximity to early COVID-19 hotspots in Northern Italy and Austria.

#### Changes in Macro- and Micro-Contexts and Income

One of the most pronounced changes in the macro- and micro-contexts beyond the household's direct control was the closure of physical workplaces. In Germany, about 30% of respondents were affected by it, in Denmark more than 40%, and in Slovenia more than 70% of the respondents were impacted. This significant difference among the three countries (*Z*-test for comparison of proportions, *p* < 0.001) is also mirrored in the number of households who experienced an income loss due to the pandemic. Overall, only 9% of Denmark's sample households experienced income loss, 23% in Germany, but more than 50% in Slovenia (*Z*-test for comparison of proportions, *p* < 0.001). Although German households reported relatively higher income gain than the other two countries, all three countries experienced substantially more income loss than income gain. In terms of national poverty data, all three countries are well below average EU28 poverty levels with only small differences between them.

#### Food Poverty and Anxiety

Table 3 also shows the changes between before and during COVID-19 reported by the sample households in terms of missed meals and anxiety about acquiring food. Regarding missed meals, there was little change between before and during in all three countries.

Regarding anxiety about acquiring food, there was significant increase from before to during (*Z*-test for comparison of proportions, *p* < 0.001), to some extent in Denmark, somewhat more in Germany and quite a lot in Slovenia.

### Changes in Food-Related Behaviors

#### Frequency of Food Shopping

Our data clearly shows that the mean frequency of food shopping significantly decreased during the pandemic compared to before (paired-samples *t*-tests, *p* < 0.001; see Supplementary Figure 1). This effect was more pronounced for fresh food compared to non-fresh food (Supplementary Figure 1). Depending on the food category, 42–58% of respondents in Slovenia reported a decrease in shopping frequency of fresh food, while 35% reported a decrease for non-fresh food. Interestingly, these numbers were significantly lower in Denmark and Germany (*Z*-tests for comparison of proportions, *p* < 0.05), where only 27–30% (DK) and 20–28% (DE) of respondents reported a decrease in shopping frequency of fresh food, and 23% (DK) and 16% (DE) for non-fresh food. In other words, the majority of respondents from Denmark and Germany did not reduce their shopping frequency.

#### Food Consumption Frequencies

The comparison of food consumption frequencies during the pandemic and before with paired-samples *t*-tests (see Supplementary Figure 2) revealed that the *mean* consumption frequencies of fresh food significantly *decreased* in the three countries, with slight variations regarding the types of food affected: fruit & vegetables—all countries; meat—all countries; fish—DE, SI; dairy—DE, DK; bread—DE, SI (all effects significant at the level *p* < 0.01 except for dairy in DK with *p* < 0.05 and dairy in DE *p* < 0.1). The consumption frequencies of non-fresh food, by contrast, significantly *increased* in Denmark and Germany in the categories of ready-made meals, sweet snacks (cake & biscuits, sweets & chocolate), and alcoholic drinks, and in Germany, the mean consumption frequency of canned food also increased (all effects significant at the level *p* < 0.01 except for sweets in DK with *p* < 0.05). In Slovenia, the mean consumption frequencies of non-fresh food did not significantly change except for ready-made meals where a significant decrease (*p* < 0.01) was observed.

**Figure 2:**
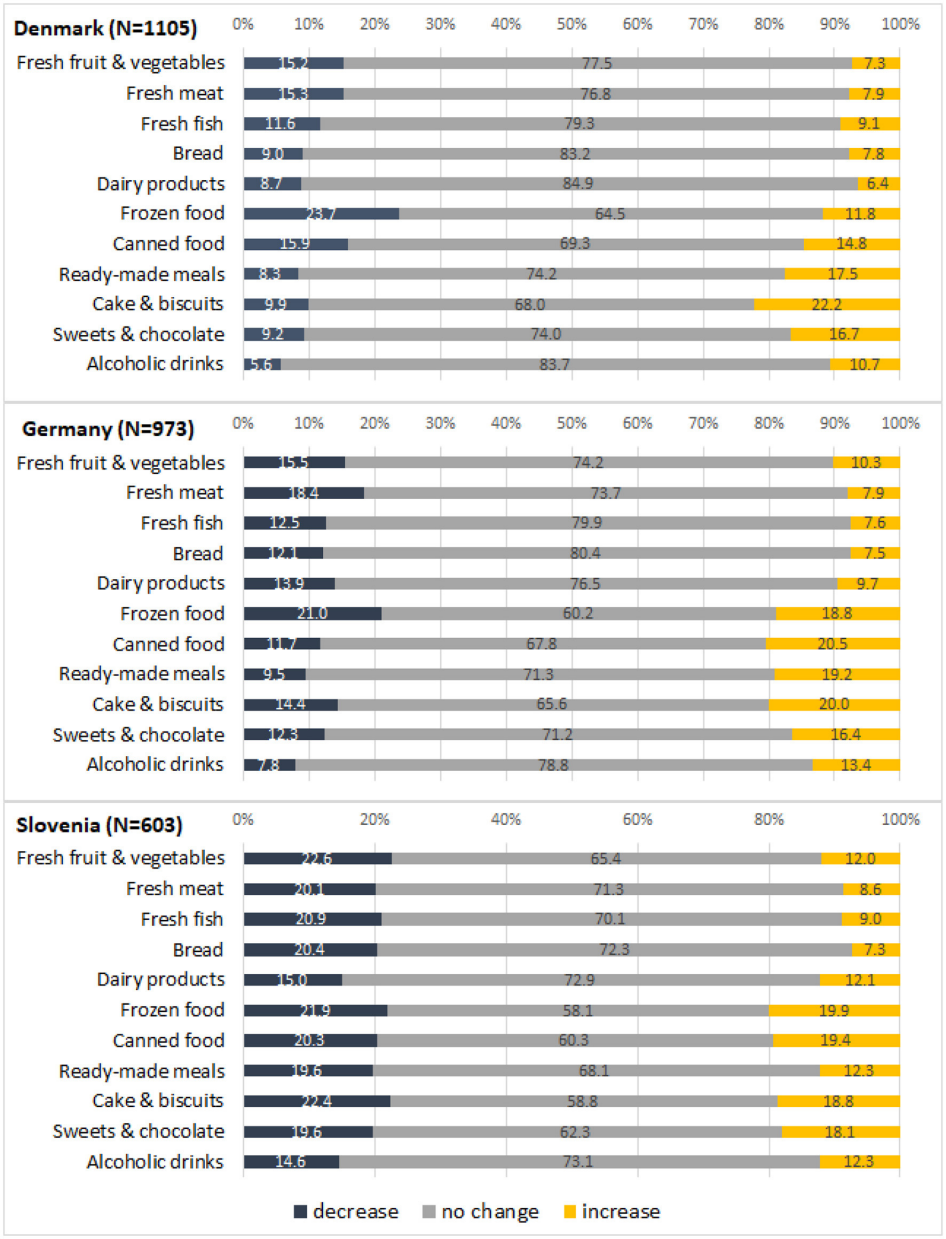
Changes in individuals' food consumption frequencies.

However, the comparison of *mean* consumption frequencies does not allow insights into the proportions of people who changed their consumption frequencies during the pandemic compared to before, and it masks the following interesting observations. When analyzing changes in consumption frequency at the individual consumer level, we observed diverging trends in all food categories. Some people decreased, others increased, and yet others did not change their consumption frequency (see Figure 2). In some categories, these diverging trends “canceled out” each other so that the mean consumption frequency did not significantly change. Our observation of diverging trends in food consumption changes are novel insights which cannot be detected by looking at aggregated data like trends in retail sales or changes in *mean* consumption frequencies.

Depending on the food category, between 15 and 42% of consumers changed their consumption frequency during the pandemic compared to before (Figure 2). Table 4 maps the changes in food consumption by category. Overall, the significantly highest proportions of people who changed consumption frequencies were observed in Slovenia (*Z*-tests for comparison of proportions, *p* < 0.05); the only exceptions were the categories frozen food and ready-made meals where the country differences were not significant (and the categories meat and cake & biscuits where the difference between Slovenia and Denmark was significant but not the difference between Slovenia and Germany).

**Table 4:** Rates of change in food consumption frequency by food category.

**Rates of change^+^**	**Denmark**	**Germany**	**Slovenia**
*High* rates of change: >30–42% of respondents	Frozen food, canned food, cake & biscuits	Frozen food, canned food, cake & biscuits	Frozen food, canned food, cake & biscuits, ready-made meals, fruit & vegetables, sweets & chocolate
*Medium* rates of change: >20–30% of respondents	Fruit & vegetables, meat, fish, ready-made meals, sweets & chocolate	Fruit & vegetables, meat, fish, ready-made meals, sweets & chocolate, dairy products, alcoholic drinks	Meat, fish, bread, dairy products, alcoholic drinks
*Low* rates of change: 15–20% of respondents	Bread, dairy products, alcoholic drinks	Bread	

+ *Percent of respondents who had changed consumption frequencies, i.e., increased or decreased consumption frequencies during the pandemic compared to before*.

Interestingly, there are great similarities between the three countries regarding the food categories with the highest and lowest rates of change (by rate of change we mean the combined proportions of people who increased or decreased their consumption). In all three countries, the highest rates of change were observed in the categories of frozen food, canned food, and cake & biscuits, while bread, dairy products, and alcoholic drinks were among the categories with the lowest rates of change (Table 4).

We also analyzed changes in consumption frequency across the 11 product categories at the individual consumer level. Interestingly, only a small proportion of respondents did not report any changes in eating frequency (15% in DK; 14% in DE; 8% in SI). About half of the respondents in Denmark and Germany and two-thirds in Slovenia reported changes in three or more product categories. Changes in five or more product categories were reported by 17% of the respondents in Denmark, 24% in Germany and 35% in Slovenia.

### Factors Related to Changes in Food Consumption Frequencies

We estimated multinomial logistic (MNL) regression models to identify factors significantly related to the observed decreases and increases in consumption frequencies in the different food categories, as outlined in Figure 2. The outcome reference category was the group of people who did not change their consumption frequency (in Figure 2 displayed in gray color). The model fit varied considerably across the different food categories (Table 5) and was generally “moderate” or “good” for fresh food, and rather “low” for non-fresh food (apart from a few exceptions). The models focused on pandemic-related factors as predictors of behavior change. It is therefore not surprising that the model fit was low in some food categories. The variance not explained by the models can be attributed to factors not controlled for, foremost differences in personal food values and strategies (such as health or convenience orientation, which were not included as predictors in the models in order to limit the predictors to a manageable number).

**Table 5:** Nagelkerke's Pseudo R-Square of the MNL regression models.

	**Denmark**	**Germany**	**Slovenia**
Fresh fruit & vegetables	0.193	0.202	0.227
Fresh meat	0.143	0.109	0.163
Fresh fish	0.159	0.209	0.174
Bread	0.321	0.223	0.249
Dairy products	0.237	0.195	0.188
Frozen food	0.120	0.174	0.098
Canned food	0.079	0.163	0.166
Ready-made meals	0.131	0.159	0.223
Cake & biscuits	0.107	0.137	0.107
Sweets & chocolate	0.067	0.113	0.092
Alcoholic drinks	0.102	0.107	0.107

The model results are summarized in Tables 6–8 (the full model results are provided in the Supplementary Tables 2–4). The remainder of the section is organized according to the independent variables analyzed in the MNL regression models. The effects mentioned in the text are significant at the level *p* < 0.01, *p* < 0.05, or *p* < 0.1 (see Tables 6–8 for level of significance).

**Table 6:**
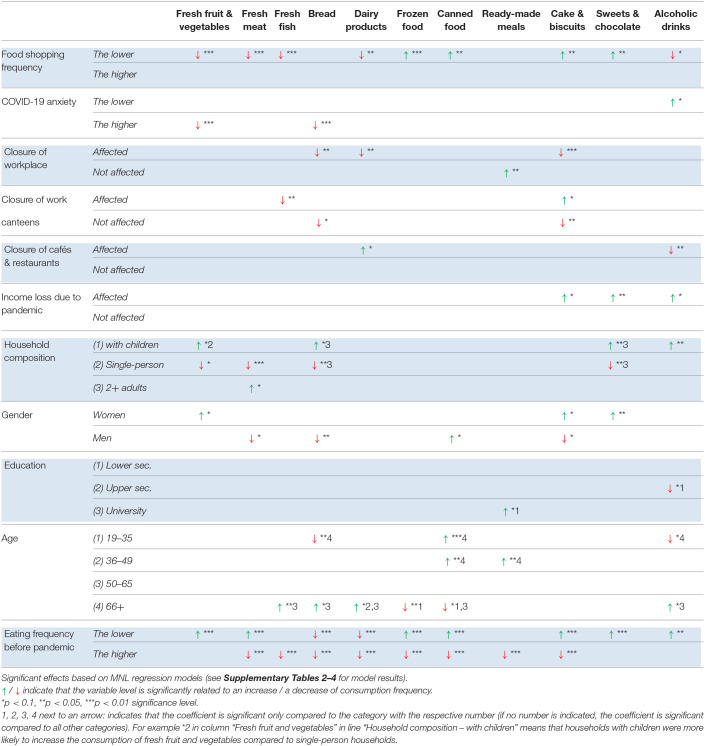
Factors significantly related to changes in food consumption frequency – DENMARK.

*Significant effects based on MNL regression models (see Supplementary Tables 2–4 for model results)*.

*↑ / ↓ indicate that the variable level is significantly related to an increase / a decrease of consumption frequency*.

^*^p < 0.1, ^**^p < 0.05, ^***^p < 0.01 significance level.

1, 2, 3, 4 next to an arrow: indicates that the coefficient is significant only compared to the category with the respective number (if no number is indicated, the coefficient is significant compared to all other categories). For example *2 in column “Fresh fruit and vegetables” in line “Household composition – with children” means that households with children were more likely to increase the consumption of fresh fruit and vegetables compared to single-person households.

**Table 7:**
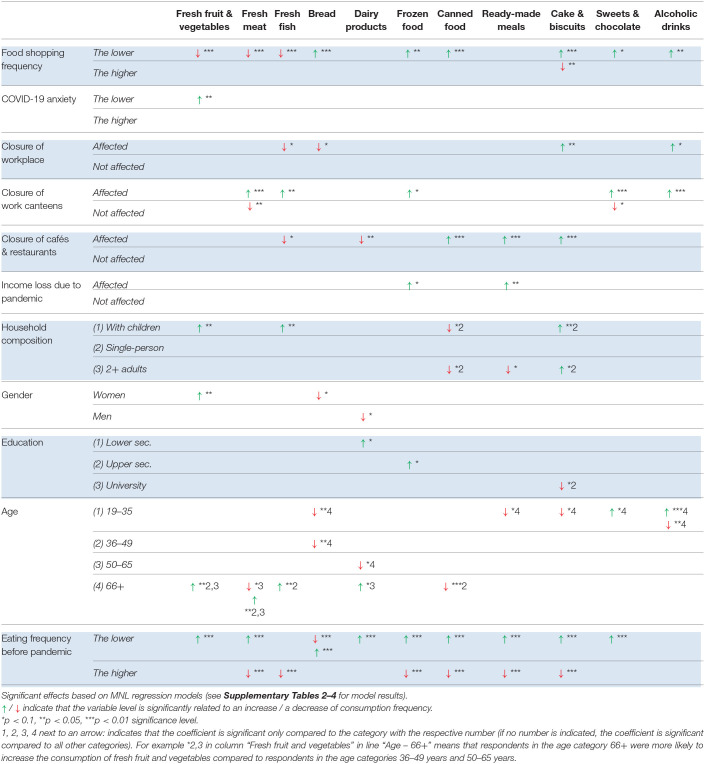
Factors significantly related to changes in food consumption frequency – GERMANY.

*Significant effects based on MNL regression models (see Supplementary Tables 2–4 for model results)*.

*↑ / ↓ indicate that the variable level is significantly related to an increase / a decrease of consumption frequency*.

^*^p < 0.1, ^**^p < 0.05, ^***^p < 0.01 significance level.

1, 2, 3, 4 next to an arrow: indicates that the coefficient is significant only compared to the category with the respective number (if no number is indicated, the coefficient is significant compared to all other categories). For example *2,3 in column “Fresh fruit and vegetables” in line “Age – 66+” means that respondents in the age category 66+ were more likely to increase the consumption of fresh fruit and vegetables compared to respondents in the age categories 36–49 years and 50–65 years.

**Table 8:**
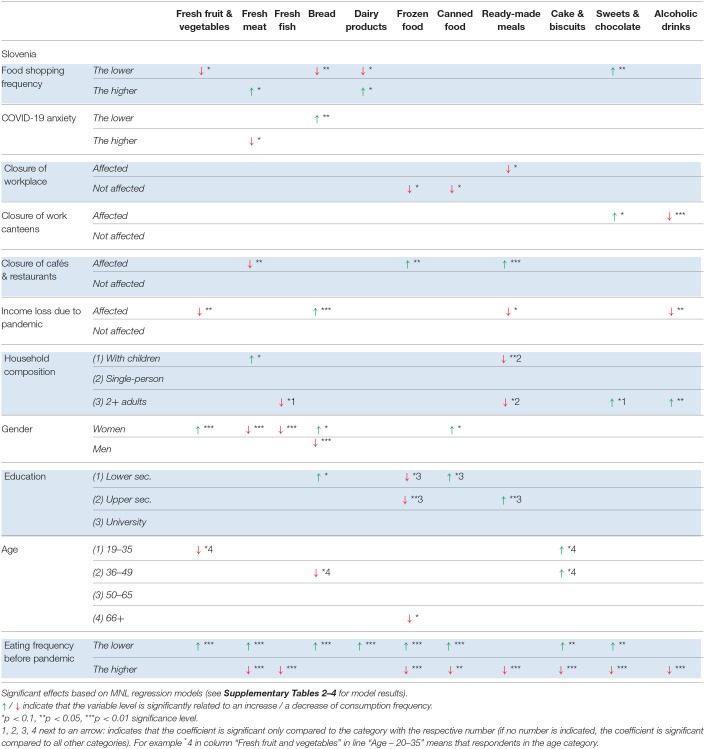
Factors significantly related to changes in food consumption frequency – SLOVENIA.

*Significant effects based on MNL regression models (see Supplementary Tables 2–4 for model results)*.

*↑ / ↓ indicate that the variable level is significantly related to an increase / a decrease of consumption frequency*.

^*^p < 0.1, ^**^p < 0.05, ^***^p < 0.01 significance level.

1, 2, 3, 4 next to an arrow: indicates that the coefficient is significant only compared to the category with the respective number (if no number is indicated, the coefficient is significant compared to all other categories). For example ^*^4 in column “Fresh fruit and vegetables” in line “Age – 20–35” means that respondents in the age category.

#### Changes in Shopping Frequency

Across the three study countries, a decrease in shopping frequency was significantly related to a decrease in fresh food consumption, with slight variations between the study countries regarding the types of fresh food affected: fruit and vegetables (all countries), meat (DE, DK), fish (DE, DK), and dairy (DK, SI). Furthermore, a decrease in shopping frequency was significantly related to an increase in frozen food and canned food consumption in Germany and Denmark, suggesting some people partly substituted fresh food with frozen food and canned food. Interestingly, a decrease in shopping frequency was also significantly related to an increase in sweet snacks in all three countries (sweets & chocolate: all countries; cake & biscuits: DE, DK).

Regarding the consumption of bread and alcohol, we observed opposite effects between the study countries. While a decrease in shopping frequency was significantly related to a decrease in bread consumption in Slovenia, it was significantly related to an increase in bread consumption in Germany. With a decrease in shopping frequency, the consumption of alcoholic drinks tended to decrease in Denmark, whereas it tended to increase in Germany.

#### COVID-19 Risk Perception

The level of perceived risk and anxiety of COVID-19 (hereafter referred to as “COVID-19 risk perception”) had significant effects on food consumption in all of the three countries, but with interesting differences between Denmark and Germany on the one hand, and Slovenia on the other hand.

In Denmark and Germany, the consumption of fresh fruit and vegetables was significantly related to COVID-19 risk perception. Higher levels of COVID-19 risk perception were associated with a decrease in the consumption of fruit and vegetables and bread in Denmark. Similarly, lower levels of COVID-19 risk perception were associated with a higher probability of increasing fruit and vegetable consumption in Germany. These trends are in contradiction to our initial assumption, according to which people who are anxious about the COVID-19 virus might try to strengthen their immune system through increased levels of fruit and vegetable consumption. We checked whether the level of perceived risk was significantly related to a change in shopping frequency and found significant correlations with small to medium effect sizes in Germany (*r* = −0.19, *p* < 0.001) and Denmark (*r* = −0.24, *p* < 0.001), but no significant correlation in Slovenia. Thus, it seems that in Germany and Denmark, people who were more anxious about the virus tended to decrease their shopping frequency even more than others did.

In Slovenia, higher levels of COVID-19 risk perception were associated with a decrease in the consumption of fresh meat, while lower levels of COVID-19 risk perception had a significant effect on the probability of increasing bread consumption.

To summarize the key results, it was *not* the case that people who were more anxious about the virus were trying to eat more healthily. On the contrary, higher levels of COVID-19 risk perception were significantly related to a decrease in fruit and vegetable consumption in Germany and Denmark. Neither was it the case that people who were more anxious about the virus increased their consumption of alcohol and sweet snacks as a means to cope with increased levels of stress. On the contrary, an increase of alcohol consumption was significantly related to *lower* levels of COVID-19 risk perception in Denmark.

#### Closure of Workplaces and Work Canteens

The proportion of respondents affected by the physical closure of their workplace differed significantly across the countries, ranging from 29% in Germany to 43% in Denmark and 74% in Slovenia. Despite this difference, the proportion of people affected by the closure of work canteens was similar across the countries: 15% (DE) respectively 17% (DK, SI) of the respondents used to eat at work canteens at least once a week before the pandemic but stopped eating there during the first lockdown. Both, the physical closure of workplaces as well as canteens had significant - and partly opposite - effects on food consumption in all of the three countries.

In Denmark, people affected by a closure of their physical workplace were more likely to decrease their consumption of bread and dairy products compared to other people. Interestingly, these people were also more likely to decrease the consumption of cake and biscuits. Another interesting phenomenon was observed regarding ready-made meals. While the consumption of ready-made meals had generally increased in Denmark during the pandemic, this effect was not seen among people affected by a lockdown of their workplace.

The closure of work canteens also had interesting effects in Denmark. People who stopped eating in canteens during the pandemic were more likely to decrease their fresh fish consumption compared to other people. Moreover, these people were more likely to increase their consumption of cake and biscuits, suggesting they substituted their canteen lunch (partly) with cake and biscuits.

Similar to the observation in Denmark, people affected by a closure of their physical workplace in Germany were also more likely to decrease their bread consumption and their consumption of fresh fish. Regarding the consumption of cake and biscuits, the effect in Germany was opposite compared to Denmark. In Germany, the consumption of cake and biscuits tended to *increase* among people affected by the lockdown of their physical workplace. Another observation was that these people were more likely to increase the consumption of alcoholic drinks.

The closure of work canteens in Germany had partly opposite effects to the closure of physical workplaces. People who had stopped eating at work canteens were more likely to *increase* fish consumption. Fish is typically served only once a week in German canteens. Furthermore, the closure of work canteens led to an increase in the consumption of frozen food, suggesting these people used frozen ingredients for preparing meals instead of going to the canteen. Moreover, people who had stopped eating at work canteens during the pandemic were likely to change their consumption of fresh meat and sweets and chocolate. Interestingly, the change could go in both directions, an increase as well as a decrease, highlighting the strong influence of contextual factors on eating patterns, however with partly opposite effects. Apparently, some people are nudged at their work canteen to eat less meat and less sweets and chocolate than they would do if they did not eat there, while other people are affected by eating at the work canteen in the opposite direction – more meat, sweets and chocolate.

In Slovenia, people affected by a lockdown of their physical workplace were more likely to reduce their consumption of ready-made meals. A decrease in the consumption of frozen food and canned food, however, was more likely among people *not* affected by a closure of their physical workplace. The closure of work canteens in Slovenia was significantly related to a decrease in alcohol consumption, but an increase in the consumption of sweets and chocolate.

#### Closure of Cafés and Restaurants

Interesting changes in food consumption were observed related to the closure of cafés and restaurants. In Germany and Slovenia, 22% of the respondents used to eat at cafés and restaurants at least once a week before (but not during) the lockdown, while in Denmark only 8% of the respondents fell into this group.

In Germany, these people were more likely to increase their consumption of ready-made meals, canned food, and cake and biscuits, and decrease their consumption of fish and dairy products, suggesting that “eating out” was substituted with convenience food and sweet snacks instead of cooking a meal from scratch. In Slovenia, a partly similar trend was observed. Here, these people were more likely to increase their consumption of ready-made meals and frozen food, while the consumption of fresh meat was more likely to decrease.

In Denmark, by contrast, people who used to eat at cafés and restaurants before the pandemic were more likely to decrease their alcohol consumption, suggesting that these people drink less alcohol when they are at home. Interestingly, such people were more likely to increase their consumption of dairy products.

#### Income Loss due to the Pandemic

The incidence of income loss due to the pandemic was very different across the three study countries (SI: 53%, DE: 23%, DK: 9%). Interestingly, the effects on changes in food consumption were also very different.

In Slovenia, a loss of income was significantly related to a decrease in the consumption of fruit and vegetables, ready-made meals, and alcoholic drinks, and an increase in the consumption of bread. Opposite trends were observed in the other two countries. In Denmark, people who had lost parts of their income were more likely to increase their consumption of sweet snacks and alcoholic drinks; in Germany, a loss of income was significantly related to an increase in the consumption of frozen food and ready-made meals.

#### Household Composition

We distinguished between three types of households: households with children, single-person households, and households with 2+ adults without children living in the household. Each of these types of households were affected by pandemic-related restrictions in different ways: households with children were affected by the lockdown of schools and day care institutions; single-person households were particularly challenged by social distancing restrictions, reduced human contact and potential feelings of loneliness; while households with 2+ adults were potentially not affected to the same extent.

We found significant effects of household composition on changes in food consumption in each country, again noting interesting differences between the countries. In Denmark, respondents living in households with children were more likely to *increase* their consumption of alcoholic drinks (compared to both other groups); sweets and chocolate, and bread (compared to households with 2+ adults); and fruit and vegetables (compared to single-person households). In Germany, households with children were more likely to *increase* the consumption of fruit and vegetables, and fish (compared to both other groups); and cake and biscuits (compared to single-person households). They were also more likely to decrease the consumption of canned food (compared to single-person households). In Slovenia, respondents from households with children were more likely to increase their consumption of fresh meat (compared to both other groups) and decrease the consumption of ready-made meals (compared to single-person households).

Respondents from households with two or more adults in Denmark were more likely to increase the consumption of fresh meat (compared to both other groups). In Germany, this group was more likely to decrease the consumption of ready-made meals (compared to both other groups); and decrease the consumption of canned food and increase the consumption of cake and biscuits (compared to single-person households). In Slovenia, respondents from households with two or more adults were more likely to *increase* the consumption of alcoholic drinks (compared to other two groups); sweets and chocolate, and fish (compared to households with children), and ready-made meals (compared to single-person households).

Respondents from single-person households in Denmark were more likely to decrease the consumption of fruit and vegetables, and fresh meat (compared to both other groups), as well as decrease the consumption of bread, and sweets and chocolate (compared to households with 2+ adults). In Germany and Slovenia, by contrast, respondents from single-person households were generally less likely to have changed their consumption frequencies compared to respondents from other types of households, indicating their food consumption patterns were less affected by the pandemic.

#### Gender

Interestingly, women were more likely to increase the consumption of fruit and vegetables than men in all three study countries. In Denmark, women were also more likely to increase the consumption of sweet snacks (sweets and chocolate, cake and biscuits), while men were more likely to decrease the consumption of cake and biscuits, fresh meat, and bread, but increase the consumption of canned food compared to women. In Germany, a decrease in the consumption of bread was more likely to occur among women than among men, while the opposite effect was found for a decrease in the consumption of dairy products. In Slovenia, women were more likely to increase the consumption of canned food and decrease the consumption of fresh meat and fish compared to men, and they were more likely to change the consumption of bread (both a decrease and an increase in bread consumption were more likely to happen among women than among men).

#### Education

We differentiated between three educational groups: lower secondary, upper secondary and equivalents, and university degree. Overall, we observed only a few significant effects. In Denmark, people with a university degree were more likely to increase the consumption of ready-made meals compared to people with a lower secondary degree. People with an upper secondary education were likely to decrease their alcohol consumption compared to people with a lower education. In Germany, people with a university degree were more likely to decrease the consumption of cake and biscuits compared to people with an upper secondary education, while the latter were more likely to increase the consumption of frozen food compared to both other population groups. People with a lower secondary degree were more likely to increase the consumption of dairy products. In Slovenia, people with a lower level of education were more likely to increase the consumption of bread compared to both other groups, and increase the consumption of canned food but decrease the consumption of frozen food compared to people with a university degree. The middle group was more likely to increase the consumption of ready-made meals and decrease the consumption of frozen food compared to people with a university degree.

#### Age

To capture non-linear relationships between the age of the respondents and changes in food consumption, we distinguished between four age groups (18–35, 36–49, 50–65, and 66+ years), and found a number of significant effects. However, it is difficult to identify patterns within and across product categories.

#### Consumption Frequencies Before the Pandemic

For each type of food, we controlled for the influence of consumption levels *before* the pandemic on the likelihood of decreasing or increasing consumption. For almost all types of food it was the case that an *increase* of consumption was more likely to happen the *lower* the consumption frequency of this type of food before the pandemic (exceptions are ready-made meals in DK and SI, alcohol in DE and SI, and fish in DK). That is, the lower the baseline consumption, the greater the probability of a reported increase during COVID-19.

For most types of food, we also observed that a *decrease* in consumption was more likely to happen when the consumption frequency was *higher* before the pandemic. That is, higher levels of baseline consumption were associated with greater probabilities of reported decreases in consumption during COVID-19. An exception was fruit and vegetables, in that a decrease in fruit and vegetable consumption was not significantly linked to the consumption level before the pandemic in any of the three countries (other exceptions are dairy products in DE and SI, sweets and chocolate in DK and DE, and alcoholic drinks in DK and DE).

Overall, these observations suggest that people's consumption levels tended to become more similar during the pandemic compared to before.

## Discussion

The main objective of the present research was to map changes in the consumption and purchase of food before and during lockdown of the COVID-19 pandemic in Denmark, Germany, and Slovenia. Broadly speaking, people across the three countries shopped less frequently during lockdown and there was an overall reduction in the consumption of fresh foods, but an increase in the consumption of food with a longer shelf life in Denmark and Germany. However, it is worth noting that within these overall trends, in all food categories there were subgroups of people that exhibited an opposite pattern of behavior, e.g., an increase in the consumption of fresh food. It is interesting that we observed changes in food consumption frequency in all food categories during the pandemic. Depending on the food category, 15 to 42% of respondents reported a change. The findings are in line with another study from Denmark (11). Only a small proportion of respondents (15% in DK; 14% in DE; 8% in SI) reported *no* change across the eleven food categories investigated. About half of the participants in Denmark and Germany and two-thirds in Slovenia reported changes in consumption frequency of three or more food categories, which represents a shift in food choice patterns. The higher rates of change in consumption frequencies in Slovenia were perhaps due to the fact that Slovenia had stricter restrictions in place compared to Germany and Denmark, and is likely to be related to the fact that greater reductions in grocery shopping frequency were reported in Slovenia than in the other two countries.

What is interesting is that differences in individual consumption levels before the pandemic tended to even out during the pandemic, perhaps because many people were facing similar conditions during the lockdown period, leading to a convergence of eating patterns, and so individual differences in macro- and micro- contexts that exerted a greater influence pre-pandemic had less of an effect during lockdown. While it is widely recognized in the literature that food choices are dynamic and evolve over the life course (28), they are also considered to be fairly stable and largely driven by habit when looking at shorter time spans. Significant shifts or turning points in food choice patterns are usually initiated by major life events such as leaving school, changing employment, or entering/leaving personal relationships (29). Our findings suggest that the COVID-19 pandemic and the related restrictions impacting on people's daily lives caused – at least temporary – shifts in food choice patterns for a large part of the population. This is an interesting finding suggesting that the COVID-19 pandemic potentially had similarly large effects on food transitions as major life events.

The question then becomes: are the observed changes a temporary phenomenon limited to the lockdown period in spring 2020, or will the changes last and become new habits? Theories on behavior change suggest that positive experiences with the behavior in question are strong drivers of enduring behavior change; negative experiences, however, are likely to have an opposite effect (24). For example, people who try a new vegetarian dish, and happen to like the taste of it, are likely to eat this food again and change their eating patterns toward more vegetarian dishes, in contrast to people who try the same dish but do not like its taste. In our study, it remains open as to what extent the respondents perceived the changes in food consumption they underwent as positive or negative experiences. It is also worth noting that *repeated exposure* to food has been proven to be a powerful driver of food choice in the literature on food choice trajectories (30). We therefore expect that food shifts experienced in the spring of 2020 will partly lead to new food habits, in particular since the COVID-19 pandemic and related restrictions will continue beyond the year 2020 in many countries around the world.

It is thus worthwhile looking at the observed changes in the different food categories in more detail. Interestingly, we were able to identify factors driving changes in individual food consumption frequency at food category level by analyzing pandemic-related contextual and personal factors as well as socio-demographic variables. The conceptual framework we developed proved very useful. The factors that were found to be associated with changes in food consumption in a more consistent pattern were decreased shopping frequency, loss of income due to the pandemic, COVID-19 risk perception, closure of physical workplaces, cafés and restaurants, having a household with children, and gender. Consumption levels before the pandemic constitute an important control variable. Overall, our findings illustrate how food choice is largely influenced by contextual factors, i.e., where and with whom people eat, and by food shopping frequency.

People tended to reduce their intake of fresh food during lockdown, particularly fruits, vegetables and meat, which is consistent with the findings of Bracale and Vaccaro (8) based on the sales data of Italian food stores. This was related to a reduction in shopping frequency in all three countries, with the greatest decrease occurring in Slovenia compared with Denmark and Germany. This could be because a government recommendation to reduce shopping frequency was in place in Slovenia but not in the other countries, together with travel restrictions (people were only allowed to travel within their municipality of residence). Moreover, it should be noted that Slovenia shares a border with Northern Italy, which was very strongly affected by COVID-19 in the spring of 2020, and this affected policy measures and, presumably, also people's behaviors. With fewer government restrictions in Germany and Denmark, we see that the reduction in shopping frequency appeared to be at least partly attributable to people's anxiety about COVID-19. That is, the greater they perceived the risk of COVID-19 to be, the less frequently they shopped (perhaps to minimize risk of infection), and thus they tended to consume less fresh produce given its perishable nature.

In Slovenia, respondents from households that had lost income due to the pandemic were more likely to decrease their fruit and vegetable intake, perhaps because income loss was much more pronounced in this country (53%) than the others (DK: 9%, DE: 23%). This household income loss in Slovenia was also associated with an increased intake of bread, and a decrease in ready-made meals and alcohol, suggesting that those who had reduced income may have shifted toward buying more affordable food.

The observed reduction in the consumption of fresh food is in contrast to the results of a study showing an increase in healthier dietary patterns (including increased intake of fruit and vegetables) in Spain during the COVID-19 confinement (13). However, it is important to note that the sample of the Spanish study comprised over 70% women (compared to the current study that was more or less representative in terms of gender) and our results show that women in all three countries were more likely to increase their intake of fruit and vegetables during lockdown. Although women generally consume more of these types of foods than do men [e.g., (31, 32)], it is interesting that this difference was further emphasized during the lockdown. This may be because women feel more strongly that eating fruit and vegetables is good for health, and they feel that they have more control over this behavior, which in turn causes them to consume more fruit and vegetables (33). Given that the pandemic likely highlighted people's health concerns, this could be why the gender difference in fruit and vegetable intake increased even more during lockdown. It remains to be seen to what extent such dietary differences may contribute to the greater severity of clinical outcomes of COVID-19 in men compared to women [e.g., (34, 35)].

Against the trend of an overall reduction in fresh foods, households with children tended to increase their intake of fresh produce in Germany during lockdown, and to a lesser extent in the other two countries. This may be due to households having to replace school lunches during lockdown.

Although people consumed more ready-made meals, sweet snacks and alcohol in Denmark and Germany during lockdown, there was no such increase in Slovenia, with even a decrease in ready-made meals. Other data from this study suggests two possible reasons for this. Firstly, because there appears to be greater anxiety in Slovenia about acquiring food than in the other two countries, and so consumers in Slovenia were less likely to increase spending on less essential food products. Consistent with this, loss of income was related to a decrease in non-essential food in Slovenia but not in Denmark nor Germany. Secondly, because households in Slovenia suffered greater income loss due to the pandemic (and so presumably had less work), participants may have had more time to prepare meals than those in Germany and Denmark.

Unsurprisingly, the closure of cafes and restaurants was associated with increased consumption of ready-made meals in Germany and Slovenia among those who had eaten out at least once a week before lockdown. Inversely, the consumption of such meals actually decreased in Germany, Slovenia, and to some extent Denmark, for those whose physical workplaces had closed. This might suggest these people had more time for meal preparation since they spent more time at home compared to their daily routine before the pandemic. However, given that there was no consistent increase in the consumption of fresh food amongst this segment, this suggests that they were not using more fresh ingredients when eating from home.

There was an overall increase in the consumption of sweet snacks in Denmark and Germany, although not in Slovenia. The rise in snack consumption is consistent with a study of Canadian middle to high income families with young children who also commonly reported consuming more snacks during the COVID-19 pandemic (36). Further, and as noted above, it is likely that the large income loss experienced in Slovenia mitigated against purchasing and eating snacks given that they tend to be a relatively expensive food item.

However, it is also worth noting that the relationship between lockdown and snacking is not always consistent – with for example, one Italian study showing an increase in the consumption of sweet snacks (9), whereas another Italian study showed a decrease in the sales of sweet snacks (8). This may partly be because people's snacking behavior may change over time the longer lockdown continues (37). Furthermore, what is considered to be a snack may not always be clear to consumers, as the same food may be a snack or part of a meal depending on when and how it is consumed.

A major strength of this study is its controlled sampling conducted over a short duration and at the same time in the three case countries. In contrast to most other studies where convenience or snowball sampling was used, the use of online panels enabled the collection of national samples which are more comparable with census data in terms of gender, age, and geography than other samples. However, our samples also exhibit deviations from census data in terms of age and education. It should be noted that strict lockdown measures were in place during data collection, so that an online study was, in practice, the only possible method. Quota sampling secured valid responses geographically across all three countries. Such an approach is, however, also subject to some limitations. For example, there was ready access to populations with internet connections but this probably mitigated against the inclusion of participants with lower socio-economic status. On the other hand, the compatibility of the survey with handheld devices should have reduced this problem to a good extent, and all three countries have relatively good internet infrastructures and digitally literate populations compared with the European average. Even so, it is likely this explains the difficulties in achieving representativeness of the study samples in terms of educational level. While in Denmark's sample the educational level is very similar to the country average, we observed slightly over-representation of higher education levels in the German and Slovenian datasets.

## Policy and Research Recommendations

The current findings suggest that it is more difficult for people to eat healthily during a COVID-19 confinement in terms of fresh fruit and vegetables. However, the fact that their reduced consumption is linked to reduced shopping frequency, and to some extent, greater risk perception of COVID-19, suggests that increasing the accessibility of such produce, such as through deliveries or pick-up points, may be a way of increasing their consumption. Although online delivery systems may be overburdened by individual orders in many cases, measures could be implemented to prioritize the more frequent delivery of more essential foods. This might include having a standard care package of food that could be produced/prepared in bulk, which could also increase the affordability of such produce. Furthermore, the delivery of such items could be prioritized by banning the delivery of non-essentials. For example, by temporarily banning the sales of bottled water, the online British supermarket chain Ocado was able to deliver to 6,000 additional homes during the pandemic (38).

The reduction in the intake of fresh fruit and vegetables during lockdown was also related to gender and loss of income. One factor that has been shown to increase fruit and vegetable intake in previous studies is the use of food banks, which provide such food at a nominal price (39, 40). Relatedly, providing fruit and vegetables for free increases their uptake among socially disadvantaged men (39) and families (41). Although our data show overall only small increases in the use of food banks and anxiety about acquiring food especially amongst participants with a lower education, such changes are significant and might be expected to be higher over a longer timeframe than the initial 5–6 weeks of the pandemic covered by this study. Differences between the three countries also show that as more households have experienced income loss, this has gone hand-in-hand with an increase in food anxiety and a greater reduction in the purchase and consumption of fresh fruit and vegetables. This suggests that those suffering from the greatest income loss shift to more affordable food and away from fresh foods, whilst those with much lower income loss instead tend to seek compensation for the huge inconveniences of lockdowns and changing routines by shifting to greater consumption of ready-made meals, sweets and alcohol, echoing the findings of a US American study among low-income adults (42). This has important policy implications, for example through the provision of greater income support for those who cannot work because of the pandemic and/or for vulnerable households that have the largest squeezes on their incomes. In terms of research, it is clear these issues also require further detailed examination.

Because women were more likely than men to increase their consumption of fruit and vegetables during lockdown, further research could examine whether such increases are due to the fact that women more strongly perceive the health benefits of these foods, and thus may increase their intake in order to support their immunity. If this is indeed the case, then perhaps increasing the awareness of the benefits of fruit and vegetables in helping to protect against COVID-19, particularly among men, may also help to increase the consumption of fruit and vegetables.

Other issues with important policy implications and that require further research include the composition of households which show important differences, for example in terms of whether or not younger children are present. Our study shows that many such households simultaneously tend to both increase their consumption of fruit and vegetables and of sweets and chocolate. These households are likely to require even more focused support than other groups, both in terms of income and of food advice, especially when they fall into the more vulnerable population categories.

Many aspects of this study have shown the importance of local and national food cultures in determining both before- and during-pandemic patterns of food purchasing, preparation and consumption and thus how people change their food behavior. Policies need to be more nuanced in taking account of these differences, working with them where they help shift people to more healthy and sustainable food, as well as attempting to mitigate their influence when they work the other way. Much more research is needed on this issue.

Finally, the results reported in this paper have revealed, as expected, the critical importance of how lockdown measures, as well as the response of the numerous actors along the food value chain, alongside food culture, have impacted moves toward or away from more healthy and sustainable diets. The paper has thrown up many unanswered questions, as well as relevant insights, so it is clear that much more research is required on these issues if policy makers are to be more successful in improving food wellbeing and reducing the negative environmental effects of the food system.

## Conclusions

Across Denmark, Germany and Slovenia people tended to reduce their consumption of fresh food, except for households with children. This change is related to reduced shopping frequency during the pandemic in all countries, and increased risk perceptions of COVID-19 in Denmark and Germany. In Denmark and Germany, people also increased their intake of non-perishable foods, but not in Slovenia, which may have been due to more people in Slovenia being affected by loss of income. Those who ate out once a week before the pandemic increased their intake of convenience foods in Germany and Slovenia during the first lockdown. Women were more likely than men to increase their intake of fresh fruit and vegetables.

The results presented here suggest that changes in eating behavior during the first wave of the pandemic were driven by contextual factors such as lockdown conditions, and personal factors such as anxiety related to COVID-19, loss of income, household composition, and gender. These results help to identify populations that are particularly vulnerable to nutritional changes during the pandemic, and potential avenues that could be explored to minimize the negative effects of the pandemic on food intake in consumers.

## Data Availability Statement

Requests to access the datasets should be directed to Jeremy Millard, jeremy.millard@3mg.org.

## Ethics Statement

The studies involving human participants were reviewed and approved by Research ethics officer, Danish Technological Institute, Aarhus, Denmark; Research ethics officer, Copenhagen Business School, Department of Management, Society and Communication, Frederiksberg, Denmark; Bioethical committee at the Higher School of Applied Sciences, Ljubljana, Slovenia. The participants provided their written informed consent to participate in this study.

## Author Contributions

MJ and HH analyzed the data with assistance from JM. All authors contributed to early drafts which were then collated, rewritten and substantially updated by MJ. All authors read and approved the final manuscript, interpreted the data, and made final revisions.

## Conflict of Interest

The authors declare that the research was conducted in the absence of any commercial or financial relationships that could be construed as a potential conflict of interest.
