# Supplementary material for: Changes in Food Consumption During the COVID-19 Pandemic: Analysis of Consumer Survey Data From the First Lockdown Period in Denmark, Germany, and Slovenia
Source: Front Nutr. 2021 Mar 8;8:635859. doi: 10.3389/fnut.2021.635859 (PMC7982667; doi:10.3389/fnut.2021.635859)
Supplement: Supplementary file 1 [file Presentation_1.pdf]

Supplementary material: Tables 1-4, Figures 1-2

Table S1: Examples of government lockdown measures in place in Denmark, Germany and Slovenia in April 2020

	**Denmark**	**Germany**	**Slovenia**
Day care institutions, Schools, Universities	All schools, kindergartens and day care institutions fully closed until April 15; primary schools and day care institutions reopened mid-April<br>Universities closed	All schools, kindergartens and day care institutions fully closed<br>Universities closed	All schools, kindergartens and day care institutions fully closed<br>Universities closed
Workplaces	All non-essential workplaces in the public sector closed<br>Private sector recommended to close or restrict numbers	Many non-essential workplaces in the public sector closed<br>Private sector recommended to close or restrict numbers	All non-essential workplaces in the public sector closed<br>Private sector recommended to close or restrict numbers
International travel	Non-essential international travel forbidden<br>Very limited international travel services	Non-essential international travel forbidden<br>Very limited international travel services	Non-essential international travel forbidden<br>Airports closed for passenger transport
National travel	No limit to personal movement<br>Public transport continued operating	Travel restrictions between and within some federal states<br>Public transport continued operating	Personal movement restricted to within own municipality<br>Shut down of all public transport (train, bus)
Restaurants	Closed (both indoor and outdoor)<br>Takeaway and delivery services still allowed	Closed (both indoor and outdoor)<br>Takeaway and delivery services still allowed	Closed (both indoor and outdoor)<br>Takeaway and delivery services still allowed
Food stores	Remained open<br>Some stores extended opening hours to ease access and spread customer numbers<br>A few stores voluntary offered exclusive access to more vulnerable people (elderly and chronically ill) for a few hours/day	Remained open<br>Mandatory use of face masks introduced on April 27	Remained open<br>Government advice to limit frequency of food purchases<br>Mandatory use of face masks<br>Closure of stores on Sundays<br>Access restricted to elderly people between 8 and 10 am
Non-essential stores	Closed until April 20	Shops smaller than 800 square metres reopened from April 20<br>Mandatory use of face masks introduced on April 27	Closed<br>Mandatory use of face masks
Private gatherings	Limited to ten persons, indoors and outdoors	Indoor gatherings forbidden, also in private homes<br>Outdoor gatherings restricted to two persons or persons from the same household	Private gatherings in public places limited to five persons (except for persons from the same household)
Other	Government advice to stay home and limit contacts	Government advice to stay home and limit contacts<br>Playgrounds closed	Government advice to stay home and limit contacts

Table S2: Multinomial logistic regression models on changes in food consumption frequencies – Denmark

Predictor variables	Model 1 Fresh fruit & vegetables	Model 2 Fresh meat	Model 3 Fresh fish	Model 4 Bread	Model 5 Dairy products	Model 6 Frozen food	Model 7 Canned food	Model 8 Ready-made meals	Model 9 Cake & biscuits	Model 10 Sweets & chocolate	Model 11 Alcoholic drinks
	Odds ratios (in brackets: 95% confidence interval)										
**Food shopping frequency**	**0.90 (0.86; 0.94)**	**0.91 (0.87; 0.95)**	0.96 (0.91; 1.02)	**0.90 (0.85; 0.94)**	**0.94 (0.88; 0.99)**	1.00 (0.96; 1.05)	0.99 (0.94; 1.04)	1.05 (0.97; 1.13)	1.03 (0.96; 1.10)	1.01 (0.94; 1.08)	**0.94 (0.88; 1.01)**
	0.96 (0.89; 1.04)	0.99 (0.92; 1.08)	0.99 (0.91; 1.08)	1.02 (0.95; 1.09)	0.98 (0.90; 1.06)	**0.92 (0.88; 0.97)**	**0.95 (0.91; 1.00)**	0.98 (0.93; 1.02)	**0.95 (0.91; 0.99)**	**0.95 (0.91; 1.00)**	0.97 (0.92; 1.03)
**Covid-19 anxiety**	**1.10 (1.02; 1.17)**	1.05 (0.98; 1.12)	**1.13 (1.04; 1.23)**	1.04 (0.96; 1.12)	1.03 (0.95; 1.12)	1.01 (0.96; 1.07)	1.01 (0.94; 1.08)	1.00 (0.91; 1.10)	1.06 (0.98; 1.15)	1.01 (0.93; 1.09)	1.04 (0.93; 1.15)
	1.04 (0.94; 1.15)	0.97 (0.88; 1.06)	0.97 (0.88; 1.08)	0.96 (0.88; 1.05)	1.02 (0.92; 1.13)	1.01 (0.94; 1.10)	1.05 (0.98; 1.13)	1.00 (0.94; 1.07)	0.96 (0.90; 1.02)	1.00 (0.94; 1.07)	**0.93 (0.86; 1.01)**
**Closure of workplace**	1.27 (0.88; 1.83)	1.17 (0.81; 1.70)	**1.67 (1.06; 2.64)**	0.98 (0.63; 1.53)	**1.81 (1.14; 2.88)**	1.03 (0.75; 1.41)	0.96 (0.66; 1.38)	1.26 (0.75; 2.11)	**2.49 (1.60; 3.88)**	1.13 (0.72; 1.78)	0.88 (0.50; 1.55)
(Affected)	1.49 (0.87; 2.56)	0.86 (0.52; 1.43)	1.58 (0.88; 2.85)	0.72 (0.45; 1.14)	1.63 (0.91; 2.93)	1.08 (0.72; 1.64)	1.10 (0.75; 1.60)	**0.66 (0.46; 0.94)**	1.03 (0.75; 1.43)	0.90 (0.63; 1.29)	1.30 (0.84; 1.99)
**Closure of work canteens**	1.09 (0.67; 1.75)	1.22 (0.76; 1.98)	**0.56 (0.29; 1.05)**	**1.77 (1.03; 3.04)**	0.92 (0.50; 1.68)	0.86 (0.56; 1.34)	1.00 (0.60; 1.66)	1.54 (0.85; 2.80)	**0.45 (0.23; 0.89)**	0.73 (0.38; 1.42)	1.11 (0.55; 2.22)
(Affected)	1.40 (0.70; 2.81)	0.94 (0.47; 1.88)	1.20 (0.60; 2.40)	0.87 (0.45; 1.66)	1.34 (0.63; 2.83)	1.01 (0.59; 1.73)	0.77 (0.46; 1.28)	1.03 (0.64; 1.64)	**1.42 (0.95; 2.13)**	1.26 (0.80; 1.97)	1.19 (0.71; 1.99)
**Closure of cafés & restaurants**	1.32 (0.73; 2.40)	1.34 (0.73; 2.47)	0.97 (0.43; 2.16)	0.50 (0.21; 1.21)	1.54 (0.74; 3.22)	1.28 (0.75; 2.19)	0.82 (0.40; 1.67)	1.11 (0.50; 2.46)	1.48 (0.72; 3.05)	1.18 (0.54; 2.60)	**2.52 (1.20; 5.26)**
(Affected)	0.96 (0.37; 2.52)	1.06 (0.47; 2.39)	1.02 (0.40; 2.59)	1.52 (0.75; 3.05)	**2.26 (0.95; 5.35)**	1.20 (0.61; 2.37)	1.57 (0.87; 2.82)	1.38 (0.78; 2.43)	1.38 (0.81; 2.35)	1.56 (0.89; 2.75)	1.46 (0.73; 2.92)
**Income loss due to pandemic**	1.07 (0.60; 1.91)	0.63 (0.31; 1.26)	1.22 (0.61; 2.44)	0.99 (0.51; 1.96)	0.92 (0.43; 1.96)	1.08 (0.65; 1.80)	0.79 (0.41; 1.53)	1.41 (0.69; 2.89)	1.62 (0.83; 3.17)	1.13 (0.52; 2.49)	1.07 (0.45; 2.55)
(Affected)	0.80 (0.33; 1.94)	1.51 (0.76; 3.01)	1.10 (0.45; 2.67)	0.75 (0.32; 1.72)	1.01 (0.44; 2.30)	0.78 (0.38; 1.59)	0.71 (0.37; 1.33)	0.81 (0.44; 1.48)	**1.51 (0.93; 2.47)**	**1.77 (1.06; 2.94)**	**1.77 (0.98; 3.19)**
**Household composition**	**1.79 (1.02; 3.14)**	**2.53 (1.43; 4.47)**	1.20 (0.65; 2.25)	1.15 (0.61; 2.16)	1.34 (0.68; 2.64)	0.87 (0.54; 1.40)	0.75 (0.44; 1.30)	1.02 (0.49; 2.10)	1.46 (0.74; 2.88)	1.10 (0.59; 2.07)	1.29 (0.58; 2.87)
(Single person)	**0.49 (0.23; 1.07)**	1.17 (0.51; 2.65)	0.55 (0.25; 1.20)	0.73 (0.38; 1.41)	1.58 (0.59; 4.28)	1.27 (0.68; 2.37)	1.21 (0.71; 2.08)	1.41 (0.85; 2.33)	0.95 (0.60; 1.50)	0.75 (0.46; 1.24)	**0.50 (0.28; 0.89)**
(Adults 2+)	1.26 (0.73; 2.16)	1.33 (0.76; 2.31)	0.70 (0.37; 1.29)	0.82 (0.45; 1.51)	0.96 (0.50; 1.87)	1.03 (0.66; 1.60)	0.75 (0.46; 1.25)	1.15 (0.58; 2.26)	1.28 (0.66; 2.45)	0.62 (0.33; 1.17)	0.94 (0.43; 2.04)
	0.75 (0.37; 1.55)	**2.02 (0.94; 4.34)**	**0.51 (0.24; 1.09)**	0.85 (0.46; 1.57)	1.74 (0.67; 4.54)	1.16 (0.63; 2.10)	0.89 (0.52; 1.52)	1.15 (0.71; 1.87)	0.78 (0.50; 1.20)	**0.54 (0.34; 0.87)**	**0.56 (0.33; 0.95)**
**Gender**	0.80 (0.55; 1.14)	**0.71 (0.50; 1.02)**	**0.57 (0.36; 0.89)**	0.77 (0.51; 1.18)	0.75 (0.48; 1.18)	0.98 (0.73; 1.33)	1.14 (0.80; 1.62)	1.41 (0.85; 2.33)	**0.66 (0.43; 1.02)**	0.81 (0.52; 1.25)	0.64 (0.37; 1.11)
(Female)	**1.63 (0.96; 2.77)**	0.82 (0.51; 1.32)	0.74 (0.43; 1.30)	1.00 (0.65; 1.55)	0.77 (0.44; 1.35)	0.86 (0.58; 1.27)	**0.71 (0.49; 1.01)**	0.81 (0.58; 1.13)	**1.30 (0.95; 1.77)**	**1.37 (0.97; 1.94)**	1.02 (0.67; 1.55)
**Age**	0.88 (0.46; 1.71)	0.78 (0.40; 1.50)	**2.42 (1.10; 5.32)**	1.02 (0.49; 2.15)	0.66 (0.27; 1.61)	**0.52 (0.30; 0.91)**	**0.33 (0.16; 0.67)**	1.90 (0.80; 4.53)	1.46 (0.69; 3.10)	0.80 (0.36; 1.79)	**2.35 (0.94; 5.88)**
(19-35)	1.01 (0.44; 2.29)	1.45 (0.65; 3.24)	1.25 (0.50; 3.10)	1.12 (0.55; 2.27)	0.71 (0.30; 1.65)	1.19 (0.62; 2.29)	**2.81 (1.51; 5.25)**	1.48 (0.83; 2.62)	1.35 (0.81; 2.27)	0.83 (0.47; 1.50)	1.14 (0.57; 2.28)
(36-49)	1.40 (0.74; 2.65)	1.20 (0.64; 2.25)	1.68 (0.73; 3.87)	0.96 (0.43; 2.13)	1.24 (0.55; 2.79)	0.77 (0.44; 1.32)	0.81 (0.43; 1.53)	2.12 (0.85; 5.30)	1.77 (0.83; 3.78)	1.21 (0.57; 2.54)	1.23 (0.44; 3.47)
	0.59 (0.22; 1.56)	1.65 (0.68; 4.02)	0.70 (0.26; 1.87)	1.21 (0.59; 2.49)	**0.25 (0.07; 0.86)**	0.93 (0.44; 1.94)	**2.30 (1.20; 4.40)**	**1.94 (1.09; 3.46)**	1.29 (0.75; 2.23)	0.95 (0.51; 1.76)	1.33 (0.66; 2.69)
(50-65)	1.27 (0.82; 1.96)	1.00 (0.65; 1.55)	1.59 (0.85; 2.97)	1.07 (0.63; 1.82)	1.35 (0.76; 2.41)	0.75 (0.52; 1.07)	**0.69 (0.45; 1.05)**	1.61 (0.79; 3.28)	1.04 (0.61; 1.76)	0.93 (0.54; 1.57)	1.19 (0.58; 2.44)
	0.63 (0.32; 1.22)	1.37 (0.75; 2.50)	**0.46 (0.21; 1.02)**	**0.55 (0.32; 0.95)**	**0.53 (0.27; 1.04)**	0.77 (0.47; 1.26)	1.41 (0.88; 2.27)	1.10 (0.73; 1.67)	0.85 (0.57; 1.25)	0.90 (0.58; 1.38)	**0.61 (0.35; 1.09)**
**Education**	0.92 (0.55; 1.52)	0.98 (0.59; 1.63)	0.97 (0.49; 1.90)	0.85 (0.44; 1.64)	1.19 (0.61; 2.32)	0.92 (0.60; 1.41)	1.37 (0.83; 2.27)	0.78 (0.38; 1.63)	1.37 (0.74; 2.51)	1.49 (0.80; 2.77)	0.44 (0.17; 1.17)
(Lower sec.)	0.53 (0.24; 1.17)	0.91 (0.45; 1.81)	1.24 (0.55; 2.83)	0.85 (0.47; 1.56)	0.98 (0.43; 2.22)	0.72 (0.40; 1.29)	0.70 (0.41; 1.22)	**0.62 (0.38; 1.02)**	1.18 (0.75; 1.85)	0.76 (0.46; 1.27)	0.63 (0.33; 1.21)
(Upper sec.)	0.81 (0.54; 1.22)	0.94 (0.62; 1.42)	0.96 (0.58; 1.58)	1.25 (0.77; 2.01)	1.12 (0.66; 1.90)	0.96 (0.68; 1.36)	1.19 (0.78; 1.79)	0.73 (0.42; 1.27)	1.04 (0.63; 1.73)	1.18 (0.70; 1.99)	1.16 (0.64; 2.09)
	0.62 (0.34; 1.11)	0.96 (0.56; 1.63)	0.66 (0.35; 1.25)	0.66 (0.41; 1.09)	0.92 (0.47; 1.80)	0.73 (0.47; 1.14)	0.87 (0.58; 1.31)	0.82 (0.57; 1.19)	1.00 (0.71; 1.42)	0.84 (0.57; 1.23)	0.70 (0.45; 1.10)
**Eating frequency before pandemic**	0.96 (0.89; 1.04)	**1.17 (1.07; 1.28)**	**0.76 (0.69; 0.84)**	**1.79 (1.56; 2.05)**	**0.80 (0.73; 0.88)**	**1.19 (1.10; 1.30)**	**1.40 (1.25; 1.58)**	**2.14 (1.79; 2.54)**	**1.24 (1.13; 1.37)**	1.02 (0.92; 1.13)	1.05 (0.93; 1.19)
	**0.54 (0.47; 0.62)**	**0.65 (0.57; 0.75)**	**0.49 (0.43; 0.55)**	0.90 (0.70; 1.15)	**0.55 (0.48; 0.62)**	**0.70 (0.60; 0.83)**	**0.57 (0.45; 0.72)**	1.07 (0.88; 1.31)	**0.85 (0.77; 0.94)**	**0.81 (0.73; 0.90)**	**0.88 (0.79; 0.99)**

Notes: (1) grey coloured rows: outcome change "decrease of consumption frequency", white coloured rows: outcome change "increase of consumption frequency". In **bold**: significant coefficient (p<0.1). (2) MNL regression variables base categories: "no change" for outcome variable consumption frequency, category "not affected" for the variables "closure of physical workplace", "closure of work canteens", "closure of cafés and restaurants", "income loss due to pandemic"; level "with children" for household composition; level "male" for gender; level "university degree" for education; level "66+" for age; (3) variables "eating frequency before pandemic", "food shopping frequency", "covid-19 anxiety" are continuous predictors.

Table S3: Multinomial logistic regression models on changes in food consumption frequencies – Germany

Predictor variables	Model 1 Fresh fruit & vegetables	Model 2 Fresh meat	Model 3 Fresh fish	Model 4 Bread	Model 5 Dairy products	Model 6 Frozen food	Model 7 Canned food	Model 8 Ready-made meals	Model 9 Cake & biscuits	Model 10 Sweets & chocolate	Model 11 Alcoholic drinks
	Odds ratios (in brackets: 95% confidence interval)										
**Food shopping frequency**	**0.88 (0.82; 0.94)**	**0.91 (0.86; 0.97)**	0.97 (0.90; 1.04)	**0.86 (0.80; 0.93)**	1.00 (0.93; 1.08)	1.04 (0.97; 1.12)	1.06 (0.97; 1.16)	1.06 (0.96; 1.16)	**0.93 (0.86; 1.00)**	1.04 (0.96; 1.13)	0.96 (0.88; 1.05)
	0.95 (0.85; 1.05)	1.07 (0.94; 1.20)	**0.88 (0.80; 0.96)**	0.95 (0.86; 1.06)	0.94 (0.85; 1.03)	**0.92 (0.86; 0.98)**	**0.91 (0.86; 0.97)**	0.97 (0.91; 1.03)	**0.89 (0.83; 0.95)**	**0.94 (0.88; 1.00)**	**0.94 (0.88; 1.00)**
**Covid-19 anxiety**	1.00 (0.93; 1.08)	0.98 (0.91; 1.05)	1.00 (0.92; 1.08)	1.06 (0.97; 1.16)	0.99 (0.92; 1.07)	0.98 (0.91; 1.04)	1.00 (0.92; 1.08)	0.97 (0.88; 1.07)	1.04 (0.96; 1.13)	1.04 (0.96; 1.12)	0.96 (0.87; 1.05)
	**0.91 (0.83; 1.00)**	0.99 (0.90; 1.09)	1.01 (0.90; 1.12)	1.01 (0.91; 1.11)	1.02 (0.93; 1.13)	0.98 (0.91; 1.05)	1.03 (0.96; 1.10)	1.05 (0.98; 1.12)	1.04 (0.97; 1.11)	1.05 (0.98; 1.13)	1.03 (0.95; 1.11)
**Closure of workplace** (Affected)	0.93 (0.61; 1.43)	0.80 (0.53; 1.22)	**1.54 (0.98; 2.41)**	**1.56 (0.95; 2.56)**	1.22 (0.79; 1.87)	1.00 (0.67; 1.50)	0.94 (0.56; 1.59)	1.34 (0.78; 2.33)	1.42 (0.92; 2.19)	1.35 (0.85; 2.14)	1.07 (0.61; 1.87)
	1.28 (0.73; 2.23)	1.05 (0.60; 1.85)	1.25 (0.68; 2.32)	1.34 (0.76; 2.35)	1.21 (0.69; 2.12)	1.04 (0.69; 1.56)	0.86 (0.58; 1.26)	0.82 (0.55; 1.22)	**1.62 (1.10; 2.39)**	1.13 (0.74; 1.71)	**1.53 (0.99; 2.35)**
**Closure of work canteens** (Affected)	1.43 (0.85; 2.39)	**1.84 (1.12; 3.03)**	1.17 (0.67; 2.05)	1.24 (0.67; 2.31)	1.05 (0.62; 1.81)	0.96 (0.57; 1.64)	0.91 (0.46; 1.80)	0.90 (0.45; 1.81)	1.18 (0.69; 2.04)	**1.68 (0.96; 2.92)**	0.88 (0.43; 1.82)
	1.24 (0.63; 2.47)	**2.53 (1.34; 4.80)**	1.42 (0.65; 3.10)	**2.09 (1.09; 3.99)**	1.41 (0.73; 2.73)	**1.65 (1.01; 2.69)**	1.12 (0.69; 1.81)	1.22 (0.75; 1.99)	1.16 (0.71; 1.90)	**2.14 (1.30; 3.52)**	**2.14 (1.31; 3.50)**
**Closure of cafés & restaurants** (Affected)	1.12 (0.72; 1.74)	1.26 (0.83; 1.89)	1.31 (0.81; 2.10)	**1.56 (0.95; 2.56)**	**1.56 (1.01; 2.43)**	1.04 (0.68; 1.59)	1.49 (0.87; 2.57)	0.96 (0.52; 1.77)	0.84 (0.51; 1.38)	1.17 (0.72; 1.90)	1.48 (0.84; 2.60)
	1.57 (0.87; 2.84)	0.97 (0.52; 1.81)	0.76 (0.38; 1.52)	0.96 (0.52; 1.78)	1.46 (0.82; 2.59)	1.28 (0.83; 1.96)	**1.97 (1.34; 2.90)**	**1.75 (1.18; 2.59)**	**1.71 (1.15; 2.54)**	1.17 (0.75; 1.83)	1.34 (0.85; 2.12)
**Income loss due to pandemic** (Affected)	1.08 (0.70; 1.67)	1.20 (0.79; 1.83)	0.91 (0.56; 1.47)	1.40 (0.86; 2.29)	1.21 (0.77; 1.88)	0.99 (0.65; 1.52)	0.79 (0.44; 1.43)	1.01 (0.55; 1.84)	1.11 (0.71; 1.75)	1.11 (0.68; 1.81)	0.66 (0.35; 1.24)
	0.85 (0.46; 1.56)	1.39 (0.77; 2.52)	0.68 (0.34; 1.33)	0.64 (0.33; 1.24)	1.12 (0.62; 2.02)	**1.48 (0.98; 2.24)**	1.36 (0.92; 2.00)	**1.57 (1.06; 2.32)**	0.90 (0.59; 1.36)	0.90 (0.58; 1.39)	1.09 (0.70; 1.71)
**Household composition** (Single person)	1.57 (0.92; 2.69)	1.09 (0.67; 1.79)	0.84 (0.47; 1.50)	0.87 (0.47; 1.61)	1.02 (0.59; 1.77)	0.81 (0.49; 1.33)	**0.54 (0.27; 1.08)**	0.97 (0.46; 2.03)	0.68 (0.39; 1.19)	1.23 (0.68; 2.22)	0.67 (0.34; 1.31)
	**0.55 (0.28; 1.10)**	0.90 (0.42; 1.91)	0.84 (0.37; 1.90)	**0.43 (0.21; 0.89)**	0.96 (0.47; 1.95)	0.98 (0.59; 1.63)	0.94 (0.58; 1.52)	0.99 (0.61; 1.60)	**0.59 (0.36; 0.98)**	0.77 (0.46; 1.30)	0.80 (0.46; 1.40)
(Adults 2+)	1.49 (0.90; 2.48)	0.99 (0.63; 1.56)	0.95 (0.57; 1.58)	0.98 (0.56; 1.71)	1.15 (0.69; 1.90)	1.12 (0.71; 1.76)	1.23 (0.69; 2.21)	**1.77 (0.91; 3.44)**	0.98 (0.60; 1.62)	1.26 (0.73; 2.20)	0.75 (0.41; 1.39)
	**0.52 (0.27; 0.98)**	1.09 (0.55; 2.16)	0.98 (0.45; 2.11)	**0.50 (0.27; 0.95)**	1.02 (0.51; 2.00)	1.18 (0.74; 1.88)	1.14 (0.73; 1.77)	0.95 (0.61; 1.49)	0.92 (0.59; 1.43)	0.93 (0.58; 1.50)	1.07 (0.66; 1.75)
**Gender** (Female)	1.02 (0.68; 1.53)	1.05 (0.72; 1.55)	**1.58 (0.98; 2.53)**	1.30 (0.81; 2.10)	**0.70 (0.46; 1.06)**	1.04 (0.72; 1.49)	0.72 (0.45; 1.16)	1.16 (0.67; 2.00)	0.70 (0.46; 1.08)	0.89 (0.57; 1.39)	0.94 (0.54; 1.63)
	**1.76 (1.04; 2.96)**	0.88 (0.51; 1.51)	0.68 (0.38; 1.20)	0.83 (0.48; 1.45)	0.77 (0.46; 1.31)	1.00 (0.68; 1.49)	0.95 (0.66; 1.36)	0.95 (0.65; 1.37)	1.10 (0.75; 1.61)	0.94 (0.63; 1.41)	1.12 (0.71; 1.76)
**Age** (19-35)	1.53 (0.76; 3.11)	0.90 (0.48; 1.66)	**2.68 (1.11; 6.46)**	0.66 (0.28; 1.53)	1.68 (0.79; 3.58)	0.82 (0.45; 1.49)	0.66 (0.31; 1.41)	**2.28 (0.97; 5.38)**	**1.89 (0.94; 3.78)**	1.43 (0.69; 2.95)	**2.78 (1.09; 7.10)**
	0.61 (0.27; 1.38)	0.60 (0.26; 1.37)	0.58 (0.21; 1.58)	0.73 (0.31; 1.72)	0.64 (0.28; 1.46)	1.28 (0.67; 2.44)	0.90 (0.48; 1.68)	1.01 (0.55; 1.85)	1.17 (0.64; 2.13)	**1.89 (0.98; 3.64)**	**3.23 (1.40; 7.41)**
(36-49)	1.69 (0.87; 3.28)	0.90 (0.50; 1.61)	**2.57 (1.08; 6.08)**	1.55 (0.72; 3.33)	1.19 (0.58; 2.48)	0.71 (0.40; 1.24)	**0.36 (0.17; 0.78)**	0.74 (0.30; 1.84)	1.03 (0.53; 2.01)	0.93 (0.46; 1.86)	1.93 (0.79; 4.70)
	**0.31 (0.13; 0.70)**	**0.47 (0.22; 1.04)**	1.20 (0.49; 2.95)	**0.31 (0.12; 0.78)**	0.66 (0.30; 1.45)	1.23 (0.67; 2.28)	1.26 (0.72; 2.23)	0.80 (0.45; 1.41)	0.65 (0.36; 1.18)	1.22 (0.65; 2.32)	1.91 (0.83; 4.39)
(50-65)	1.54 (0.84; 2.84)	**0.62 (0.36; 1.05)**	1.78 (0.78; 4.05)	1.41 (0.70; 2.82)	**1.77 (0.93; 3.35)**	0.83 (0.51; 1.35)	0.81 (0.45; 1.48)	1.08 (0.49; 2.38)	0.88 (0.48; 1.60)	0.90 (0.49; 1.67)	1.17 (0.51; 2.71)
	**0.40 (0.20; 0.82)**	**0.41 (0.21; 0.83)**	0.70 (0.29; 1.70)	0.61 (0.29; 1.25)	**0.50 (0.24; 1.06)**	0.82 (0.47; 1.43)	0.95 (0.57; 1.60)	0.78 (0.47; 1.29)	0.72 (0.43; 1.21)	0.99 (0.55; 1.75)	1.75 (0.81; 3.81)
**Education** (Lower sec.)	1.36 (0.71; 2.62)	0.89 (0.46; 1.74)	1.50 (0.69; 3.29)	1.00 (0.46; 2.17)	0.96 (0.45; 2.04)	0.88 (0.48; 1.59)	0.69 (0.32; 1.49)	1.02 (0.42; 2.50)	0.81 (0.41; 1.62)	1.57 (0.79; 3.10)	0.86 (0.34; 2.21)
	1.33 (0.59; 2.98)	1.30 (0.58; 2.95)	1.62 (0.61; 4.32)	0.69 (0.27; 1.79)	**2.13 (0.97; 4.70)**	0.80 (0.40; 1.62)	1.00 (0.54; 1.85)	1.32 (0.74; 2.36)	0.92 (0.50; 1.71)	1.00 (0.51; 1.95)	1.17 (0.54; 2.53)
(Upper sec.)	1.16 (0.78; 1.75)	1.19 (0.81; 1.74)	1.21 (0.77; 1.91)	1.12 (0.69; 1.81)	1.36 (0.90; 2.07)	1.10 (0.76; 1.59)	0.97 (0.60; 1.56)	0.83 (0.49; 1.40)	**0.68 (0.45; 1.02)**	1.08 (0.69; 1.69)	0.84 (0.50; 1.42)
	0.93 (0.54; 1.62)	1.19 (0.69; 2.04)	1.06 (0.58; 1.95)	0.89 (0.53; 1.52)	1.04 (0.60; 1.81)	**1.44 (0.97; 2.13)**	1.15 (0.80; 1.65)	1.08 (0.74; 1.56)	0.96 (0.66; 1.40)	1.10 (0.74; 1.65)	1.22 (0.79; 1.88)
**Eating frequency before pandemic**	0.95 (0.87; 1.04)	**1.28 (1.16; 1.42)**	**0.86 (0.79; 0.94)**	**2.62 (2.07; 3.31)**	0.96 (0.88; 1.04)	**1.45 (1.28; 1.64)**	**1.81 (1.52; 2.16)**	**2.14 (1.76; 2.61)**	**1.15 (1.05; 1.26)**	1.02 (0.94; 1.12)	1.10 (0.98; 1.23)
	**0.56 (0.49; 0.63)**	**0.80 (0.65; 0.97)**	**0.54 (0.48; 0.62)**	1.07 (0.74; 1.54)	**0.57 (0.51; 0.65)**	**0.48 (0.38; 0.60)**	**0.55 (0.43; 0.70)**	**0.77 (0.60; 0.99)**	**0.76 (0.68; 0.86)**	**0.71 (0.64; 0.79)**	0.92 (0.82; 1.03)

Notes: (1) grey coloured rows: outcome change ″decrease of consumption frequency″, white coloured rows: outcome change ″increase of consumption frequency″. In **bold**: significant coefficient (p<0.1). (2) MNL regression variables base categories: "no change" for outcome variable consumption frequency, category ″not affected″ for the variables "closure of physical workplace", "closure of work canteens", "closure of cafés and restaurants", "income loss due to pandemic"; level "with children" for household composition; level "male" for gender; level "university degree" for education; level "66+" for age; (3) variables "eating frequency before pandemic", "food shopping frequency", "covid-19 anxiety" are continuous predictors.

Table S4: Multinomial logistic regression models on changes in food consumption frequencies – Slovenia

Predictor variables	Model 1 Fresh fruit & vegetables	Model 2 Fresh meat	Model 3 Fresh fish	Model 4 Bread	Model 5 Dairy products	Model 6 Frozen food	Model 7 Canned food	Model 8 Ready-made meals	Model 9 Cake & biscuits	Model 10 Sweets & chocolate	Model 11 Alcoholic drinks
	Odds ratios (in brackets: 95% confidence interval)										
**Food shopping frequency**	**0.96 (0.92; 1.01)**	1.01 (0.96; 1.06)	**0.94 (0.90; 0.99)**	0.98 (0.94; 1.03)	**0.96 (0.91; 1.01)**	1.00 (0.95; 1.05)	0.99 (0.94; 1.04)	1.03 (0.97; 1.09)	0.99 (0.94; 1.04)	0.98 (0.93; 1.03)	1.01 (0.95; 1.07)
	0.97 (0.91; 1.03)	**1.07 (0.99; 1.16)**	0.95 (0.86; 1.04)	1.01 (0.94; 1.08)	**1.07 (0.99; 1.15)**	0.97 (0.92; 1.02)	0.97 (0.93; 1.02)	0.97 (0.91; 1.02)	0.98 (0.93; 1.03)	**0.95 (0.90; 1.00)**	0.96 (0.91; 1.02)
**Covid-19 anxiety**	1.05 (0.98; 1.14)	**1.07 (0.99; 1.16)**	0.99 (0.91; 1.07)	1.04 (0.96; 1.13)	1.04 (0.95; 1.13)	1.04 (0.96; 1.13)	1.06 (0.97; 1.14)	1.05 (0.96; 1.14)	0.98 (0.91; 1.06)	1.05 (0.97; 1.14)	1.03 (0.94; 1.12)
	0.99 (0.88; 1.10)	1.03 (0.91; 1.16)	**0.85 (0.74; 0.97)**	0.99 (0.89; 1.11)	1.06 (0.95; 1.18)	1.01 (0.93; 1.10)	1.04 (0.96; 1.13)	0.99 (0.90; 1.09)	1.00 (0.92; 1.09)	1.03 (0.95; 1.12)	1.01 (0.92; 1.11)
**Closure of workplace** (Affected)	1.04 (0.65; 1.67)	1.26 (0.76; 2.07)	1.05 (0.65; 1.71)	1.07 (0.65; 1.77)	0.72 (0.43; 1.19)	**0.64 (0.40; 1.02)**	**0.65 (0.41; 1.04)**	**1.71 (0.97; 3.03)**	1.08 (0.67; 1.74)	1.04 (0.64; 1.70)	1.53 (0.85; 2.75)
	0.69 (0.37; 1.27)	1.25 (0.61; 2.57)	0.57 (0.26; 1.25)	1.02 (0.53; 1.97)	1.36 (0.71; 2.59)	1.05 (0.64; 1.74)	0.75 (0.46; 1.23)	1.10 (0.62; 1.96)	1.07 (0.65; 1.75)	1.00 (0.60; 1.66)	1.03 (0.57; 1.84)
**Closure of work canteens** (Affected)	1.07 (0.62; 1.84)	1.11 (0.61; 2.02)	1.52 (0.87; 2.67)	1.00 (0.54; 1.86)	1.21 (0.63; 2.31)	0.98 (0.54; 1.81)	0.69 (0.37; 1.32)	1.25 (0.69; 2.26)	1.29 (0.73; 2.29)	1.14 (0.62; 2.08)	**2.30 (1.24; 4.27)**
	1.35 (0.60; 3.01)	1.20 (0.55; 2.61)	2.05 (0.79; 5.32)	1.07 (0.50; 2.32)	1.40 (0.64; 3.07)	0.68 (0.36; 1.28)	0.93 (0.51; 1.69)	0.73 (0.34; 1.57)	1.41 (0.79; 2.52)	**1.67 (0.93; 2.99)**	1.51 (0.77; 2.95)
**Closure of cafés & restaurants** (Affected)	1.46 (0.87; 2.44)	**1.79 (1.03; 3.11)**	0.78 (0.45; 1.36)	1.39 (0.78; 2.45)	0.87 (0.46; 1.66)	0.86 (0.47; 1.56)	0.74 (0.40; 1.35)	1.40 (0.78; 2.51)	1.17 (0.67; 2.02)	1.18 (0.67; 2.07)	0.68 (0.35; 1.31)
	1.28 (0.60; 2.75)	1.50 (0.71; 3.15)	1.45 (0.60; 3.49)	0.77 (0.35; 1.67)	1.12 (0.54; 2.33)	**1.84 (1.05; 3.20)**	1.48 (0.86; 2.55)	**2.36 (1.26; 4.40)**	1.43 (0.82; 2.47)	1.57 (0.89; 2.74)	1.37 (0.73; 2.57)
**Income loss due to pandemic** (Affected)	**1.74 (1.13; 2.68)**	1.18 (0.76; 1.83)	1.12 (0.72; 1.73)	1.39 (0.87; 2.20)	0.92 (0.57; 1.51)	1.04 (0.67; 1.63)	1.37 (0.88; 2.15)	**1.56 (0.95; 2.55)**	1.11 (0.72; 1.72)	1.12 (0.72; 1.76)	**1.67 (1.00; 2.78)**
	1.06 (0.59; 1.90)	0.95 (0.50; 1.83)	**5.17 (2.17; 12.31)**	1.05 (0.57; 1.93)	0.99 (0.56; 1.75)	1.42 (0.90; 2.25)	1.05 (0.67; 1.66)	0.86 (0.50; 1.47)	1.20 (0.76; 1.90)	0.87 (0.55; 1.38)	1.57 (0.91; 2.71)
**Household composition** (Single person)	0.74 (0.31; 1.78)	1.61 (0.72; 3.61)	1.50 (0.65; 3.48)	0.69 (0.24; 2.01)	0.77 (0.29; 2.07)	0.70 (0.30; 1.62)	0.95 (0.40; 2.25)	**0.27 (0.08; 0.92)**	1.14 (0.52; 2.52)	1.28 (0.55; 2.99)	0.80 (0.28; 2.33)
	1.33 (0.45; 3.93)	**0.22 (0.05; 1.07)**	0.82 (0.19; 3.49)	0.92 (0.31; 2.74)	1.17 (0.42; 3.23)	0.76 (0.30; 1.92)	1.60 (0.70; 3.64)	1.66 (0.65; 4.22)	1.03 (0.43; 2.48)	1.30 (0.54; 3.15)	0.46 (0.10; 2.10)
(Adults 2+)	0.97 (0.62; 1.52)	1.02 (0.63; 1.67)	1.48 (0.92; 2.39)	**1.52 (0.93; 2.49)**	0.87 (0.52; 1.48)	0.80 (0.49; 1.29)	0.97 (0.60; 1.57)	0.87 (0.53; 1.45)	0.84 (0.52; 1.34)	1.25 (0.77; 2.03)	1.39 (0.81; 2.38)
	1.36 (0.72; 2.57)	**0.55 (0.28; 1.09)**	0.96 (0.42; 2.17)	0.78 (0.40; 1.48)	1.06 (0.56; 2.01)	1.22 (0.75; 1.99)	1.27 (0.77; 2.07)	1.48 (0.82; 2.67)	0.98 (0.60; 1.59)	**1.56 (0.94; 2.58)**	**2.12 (1.20; 3.77)**
**Gender** (Female)	0.93 (0.62; 1.42)	**1.92 (1.24; 2.97)**	**1.91 (1.24; 2.94)**	**1.85 (1.17; 2.91)**	1.26 (0.79; 2.02)	1.23 (0.80; 1.89)	1.06 (0.69; 1.63)	0.77 (0.48; 1.24)	1.26 (0.82; 1.91)	1.20 (0.78; 1.86)	0.91 (0.54; 1.51)
	**2.02 (1.12; 3.64)**	1.47 (0.77; 2.81)	**1.98 (0.92; 4.24)**	1.07 (0.58; 1.95)	1.54 (0.88; 2.69)	1.23 (0.79; 1.93)	**1.55 (0.99; 2.44)**	0.69 (0.41; 1.18)	1.21 (0.77; 1.89)	1.37 (0.87; 2.16)	1.07 (0.62; 1.85)
**Age** (19-35)	**3.06 (0.84; 11.08)**	0.56 (0.22; 1.46)	2.89 (0.79; 10.65)	0.99 (0.35; 2.82)	0.92 (0.27; 3.10)	**0.34 (0.13; 0.87)**	0.65 (0.25; 1.70)	0.83 (0.29; 2.42)	0.77 (0.31; 1.94)	1.04 (0.38; 2.82)	0.71 (0.25; 1.98)
	2.18 (0.41; 11.62)	4.01 (0.48; 33.87)	1.01 (0.08; 12.86)	5.66 (0.69; 46.35)	0.74 (0.19; 2.85)	1.35 (0.43; 4.25)	2.01 (0.60; 6.68)	3.69 (0.76; 17.80)	**7.45 (0.94; 58.94)**	1.99 (0.60; 6.58)	4.40 (0.55; 35.56)
(36-49)	2.29 (0.64; 8.21)	0.51 (0.20; 1.30)	**3.59 (1.00; 12.90)**	1.09 (0.39; 3.01)	1.51 (0.47; 4.91)	**0.46 (0.19; 1.13)**	0.87 (0.35; 2.17)	0.71 (0.25; 2.03)	0.70 (0.29; 1.72)	0.85 (0.32; 2.28)	0.67 (0.24; 1.81)
	1.19 (0.22; 6.31)	1.58 (0.18; 13.64)	0.74 (0.06; 9.64)	3.21 (0.39; 26.26)	0.94 (0.25; 3.49)	1.11 (0.36; 3.43)	1.95 (0.59; 6.39)	3.12 (0.66; 14.82)	**7.64 (0.98; 59.74)**	1.91 (0.59; 6.21)	4.16 (0.52; 33.37)
(50-65)	2.64 (0.75; 9.28)	0.74 (0.31; 1.78)	1.74 (0.49; 6.20)	0.85 (0.32; 2.29)	1.24 (0.39; 3.92)	**0.42 (0.18; 1.00)**	0.68 (0.28; 1.64)	0.62 (0.22; 1.72)	0.68 (0.29; 1.60)	0.87 (0.34; 2.24)	0.59 (0.22; 1.56)
	2.69 (0.53; 13.61)	1.99 (0.24; 16.49)	1.16 (0.09; 14.18)	2.13 (0.26; 17.43)	1.32 (0.37; 4.72)	1.05 (0.35; 3.12)	1.35 (0.42; 4.31)	2.37 (0.52; 10.91)	**5.47 (0.70; 42.59)**	1.59 (0.50; 5.05)	3.46 (0.44; 27.29)
**Education** (Lower sec.)	1.61 (0.56; 4.63)	0.23 (0.03; 1.82)	1.49 (0.52; 4.24)	0.72 (0.19; 2.76)	1.99 (0.65; 6.11)	**2.43 (0.87; 6.76)**	1.29 (0.41; 4.03)	1.18 (0.34; 4.06)	1.53 (0.53; 4.43)	1.37 (0.49; 3.87)	1.22 (0.37; 4.00)
	1.42 (0.35; 5.76)	2.23 (0.58; 8.50)	**4.71 (0.88; 25.12)**	0.84 (0.17; 4.12)	0.96 (0.23; 4.01)	1.02 (0.26; 3.94)	**2.47 (0.87; 7.04)**	1.46 (0.37; 5.70)	1.55 (0.50; 4.79)	0.50 (0.11; 2.35)	0.71 (0.15; 3.43)
(Upper sec.)	1.27 (0.82; 1.99)	1.02 (0.65; 1.61)	0.90 (0.57; 1.40)	1.41 (0.87; 2.27)	1.30 (0.77; 2.19)	**1.70 (1.05; 2.76)**	1.17 (0.74; 1.87)	1.51 (0.91; 2.51)	1.06 (0.68; 1.66)	1.06 (0.67; 1.69)	0.95 (0.57; 1.59)
	1.23 (0.65; 2.30)	1.61 (0.80; 3.25)	1.11 (0.50; 2.48)	1.18 (0.62; 2.23)	**0.62 (0.35; 1.10)**	1.19 (0.75; 1.90)	1.14 (0.71; 1.83)	**1.89 (1.05; 3.43)**	1.29 (0.80; 2.08)	1.36 (0.84; 2.22)	1.29 (0.73; 2.27)
**Eating frequency before pandemic**	0.99 (0.88; 1.11)	**1.18 (1.06; 1.32)**	0.94 (0.86; 1.03)	**2.17 (1.69; 2.78)**	0.94 (0.85; 1.03)	**1.45 (1.26; 1.67)**	**1.18 (1.03; 1.35)**	**2.16 (1.72; 2.72)**	**1.30 (1.17; 1.46)**	**1.19 (1.08; 1.32)**	**1.17 (1.04; 1.32)**
	**0.54 (0.46; 0.63)**	**0.70 (0.58; 0.85)**	**0.54 (0.45; 0.64)**	0.92 (0.57; 1.49)	**0.61 (0.54; 0.70)**	**0.62 (0.48; 0.80)**	**0.73 (0.59; 0.89)**	0.86 (0.58; 1.30)	**0.83 (0.69; 0.99)**	**0.85 (0.74; 0.98)**	0.92 (0.78; 1.09)

Notes: (1) grey coloured rows: outcome change ″decrease of consumption frequency″, white coloured rows: outcome change ″increase of consumption frequency″. In **bold**: significant coefficient ($p<0.1$). (2) MNL regression variables base categories: "no change" for outcome variable consumption frequency, category ″not affected″ for the variables "closure of physical workplace", "closure of work canteens", "closure of cafés and restaurants", "income loss due to pandemic"; level "with children" for household composition; level "male" for gender; level "university degree" for education; level "66+" for age; (3) variables "eating frequency before pandemic", "food shopping frequency", "covid-19 anxiety" are continuous predictors.

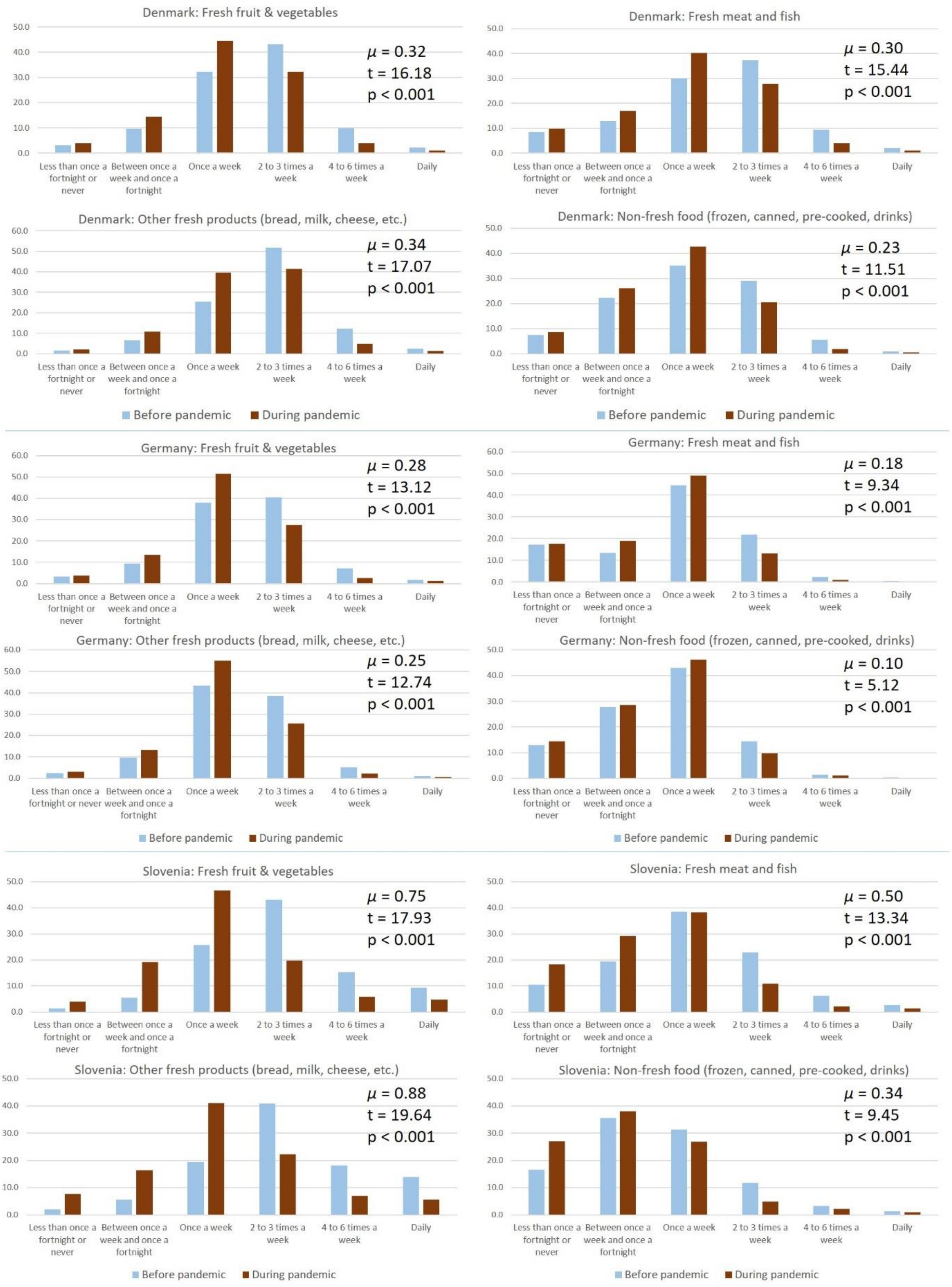

Figure S1: Shopping frequencies before and during the COVID-19 pandemic

Notes: Comparisons of mean shopping frequencies before and during the pandemic are based on paired-samples t-tests. The letter μ refers to the mean difference.

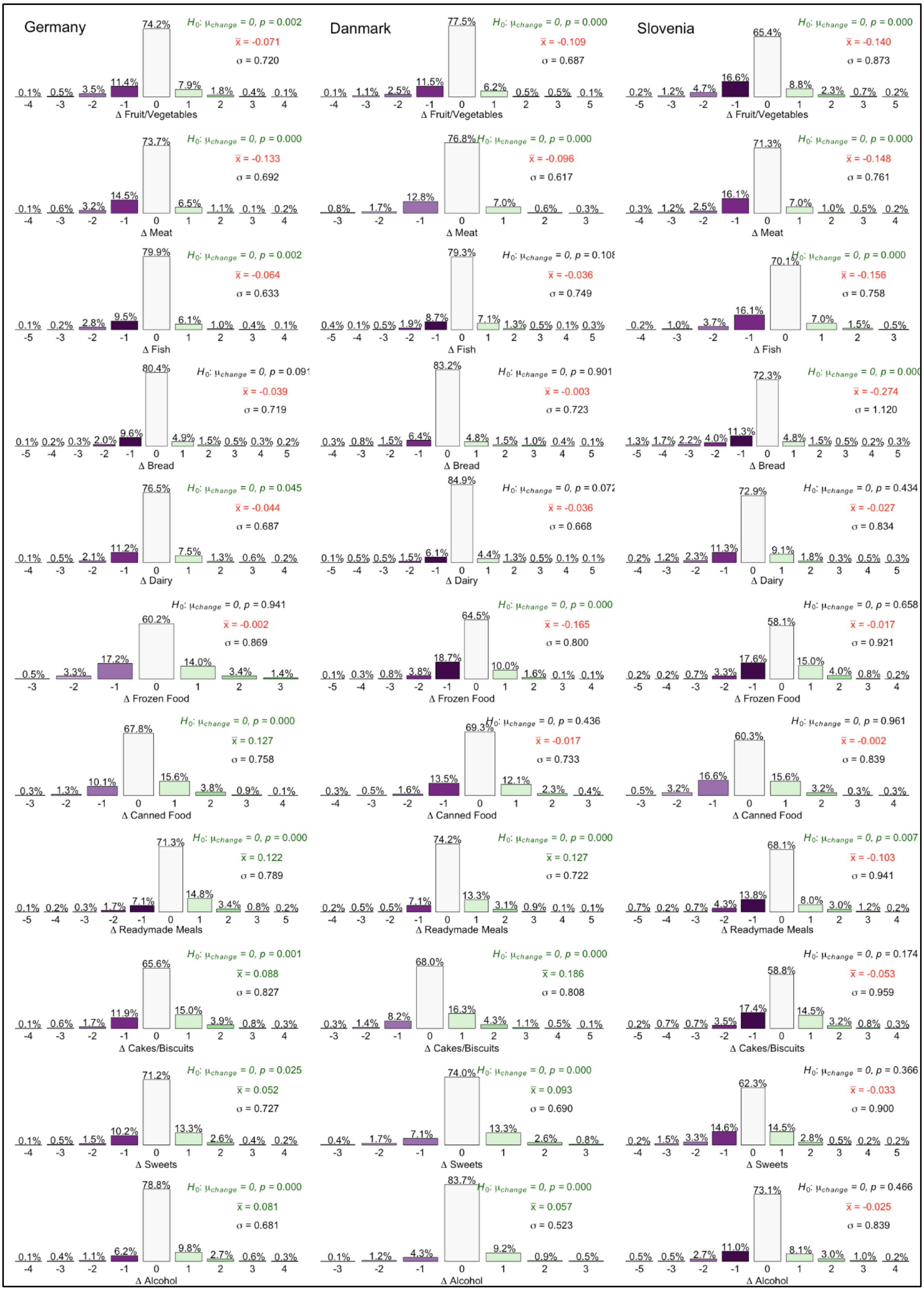

Figure S2: Changes in food consumption frequencies during the COVID-19 pandemic compared to before

Notes: Comparisons of mean consumption frequencies are based on paired-samples t-tests.
