# Supplementary material for: Changes in Food Consumption During the COVID-19 Pandemic: Analysis of Consumer Survey Data From the First Lockdown Period in Denmark, Germany, and Slovenia
Source: Front Nutr. 2021 Mar 8;8:635859. doi: 10.3389/fnut.2021.635859 (PMC7982667; doi:10.3389/fnut.2021.635859)
Supplement: Supplementary file 2 [file Presentation_2.pdf]

OUR RELATIONSHIP WITH FOOD DURING THE COVID-19 PANDEMIC

This short European-wide survey is designed to capture important aspects of your behavior and attitudes to food during this time of pandemic. Your participation will be highly appreciated and help us to understand how food systems, as a central aspect of our lives, can be improved for everyone's benefit over the longer term.

The questionnaire takes about **10-15 minutes** to complete.

Unless where clearly stated otherwise, please answer on behalf of all the people in or connected to your household who are typically involved together in obtaining, preparing and eating food, not just yourself as an individual. If there is someone else in your household who has more knowledge than you about some of the questions, please feel free to ask her or him. Please ensure that only one response is submitted per household.

Please note:

- The results of this research will be used for scientific purposes only and may be published.
- All data will be treated anonymously and confidentially at every stage of the research.
- Only general statistics will appear in reports.
- This questionnaire is 100% compliant with the EU's GDPR regulations. Please click here for more information.
- As an independent research initiative, it is entirely free from any political, commercial or vested interest.
- You have the right to discontinue the survey at any time.

◯ **I understand and accept the points stated above and wish to participate in this study.**

Seite 05

start

In the following questions, we are interested in your behaviour both before and during the Covid-19 pandemic.

By the term during Covid-19, we mean the situation right now.

Seite 06

obtain

1. What are the main ways your household´s food is obtained before and during Covid-19?

Click as many options as are relevant!

Before Covid-19

- ☐ Own purchase
- ☐ Food banks, charities
- ☐ Grow own
- ☐ Other (please specify): ______

During Covid-19

- ☐ Own purchase
- ☐ Food banks, charities
- ☐ Grow own
- ☐ Other (please specify): ______

2. What are the main sources of buying your household´s FRESH VEGETABLES / FRUITS before and during Covid-19?

Click as many options as are relevant!

Before Covid-19

- ☐ Big supermarkets
- ☐ Supermarkets
- ☐ Discount shops
- ☐ Bakeries, butchers, small grocery shops
- ☐ Organic shops
- ☐ Farm markets or street markets
- ☐ Cooperatively owned or solidarity shops
- ☐ Direct from local producer
- ☐ Home delivery (ordered online, by phone or in-person)
- ☐ Click this point to confirm you are not a robot
- ☐ Other (please specify): ______

During Covid-19

- ☐ Big supermarkets
- ☐ Supermarkets
- ☐ Discount shops
- ☐ Bakeries, butchers, small grocery shops
- ☐ Organic shops
- ☐ Farm markets or street markets
- ☐ Cooperatively owned or solidarity shops
- ☐ Direct from local producer
- ☐ Home delivery (ordered online, by phone or in-person)
- ☐ Other (please specify): ______

3. What are the main sources of buying your household´s OTHER FRESH PRODUCTS (MEAT, FISH, BREAD, MILK, CHEESE,ETC) before and during Covid-19?

Click as many options as are relevant!

Before Covid-19

- ☐ Big supermarkets
- ☐ Supermarkets
- ☐ Discount shops
- ☐ Bakeries, butchers, small grocery shops
- ☐ Organic shops
- ☐ Farm markets or street markets
- ☐ Cooperatively owned or solidarity shops
- ☐ Direct from local producer
- ☐ Home delivery (ordered online, by phone or in-person)
- ☐ Other (please specify): ______

During Covid-19

- ☐ Big supermarkets
- ☐ Supermarkets
- ☐ Discount shops
- ☐ Bakeries, butchers, small grocery shops
- ☐ Organic shops
- ☐ Farm markets or street markets
- ☐ Cooperatively owned or solidarity shops
- ☐ Direct from local producer
- ☐ Home delivery (ordered online, by phone or in-person)
- ☐ Other (please specify): ______

4. What are the main sources of buying your household´s NON-FRESH FOOD(FROZEN, CANNEND, PRE-COOKED, DRINKS, ETC) before and during Covid-19?

Click as many options as are relevant!

Before Covid-19

- ☐ Big supermarkets
- ☐ Supermarkets
- ☐ Discount shops
- ☐ Bakeries, butchers, small grocery shops
- ☐ Organic shops
- ☐ Farm markets or street markets
- ☐ Cooperatively owned or solidarity shops
- ☐ Direct from local producer
- ☐ Home delivery (ordered online, by phone or in-person)
- ☐ Other (please specify): ______

During Covid-19

- ☐ Big supermarkets
- ☐ Supermarkets
- ☐ Discount shops
- ☐ Bakeries, butchers, small grocery shops
- ☐ Organic shops
- ☐ Farm markets or street markets
- ☐ Cooperatively owned or solidarity shops
- ☐ Direct from local producer
- ☐ Home delivery (ordered online, by phone or in-person)
- ☐ Other (please specify): ______

5. How often does your household typically obtain the following food types before and during Covid-19?

Before Covid-19

Fresh vegetables and fruit

Daily
4 to 6 times a week
2 to 3 times a week
Once a week
Between once a week and once a fortnight
Less than once a fortnight or never

Fresh meat and fish

Daily
4 to 6 times a week
2 to 3 times a week
Once a week
Between once a week and once a fortnight
Less than once a fortnight or never

Other fresh products (bread, milk, cheese, etc.)

Daily
4 to 6 times a week
2 to 3 times a week
Once a week
Between once a week and once a fortnight
Less than once a fortnight or never

Non-fresh food (frozen, canned, pre-cooked, drinks, etc)

Daily
4 to 6 times a week
2 to 3 times a week
Once a week
Between once a week and once a fortnight
Less than once a fortnight or never

Other (please specify):

Daily
4 to 6 times a week
2 to 3 times a week
Once a week
Between once a week and once a fortnight
Less than once a fortnight or never

During Covid-19

Fresh vegetables and fruit

Daily
4 to 6 times a week
2 to 3 times a week
Once a week
Between once a week and once a fortnight
Less than once a fortnight or never

Fresh meat and fish

Daily
4 to 6 times a week
2 to 3 times a week
Once a week
Between once a week and once a fortnight
Less than once a fortnight or never

Other fresh products (bread, milk, cheese, etc.)

Daily
4 to 6 times a week
2 to 3 times a week
Once a week
Between once a week and once a fortnight
Less than once a fortnight or never

Non-fresh food (frozen, canned, pre-cooked, drinks, etc)

Daily
4 to 6 times a week
2 to 3 times a week
Once a week
Between once a week and once a fortnight
Less than once a fortnight or never

Other (please specify):

Daily
4 to 6 times a week
2 to 3 times a week
Once a week
Between once a week and once a fortnight
Less than once a fortnight or never

6. Which meals are typically prepared and eaten at home by at least one member of your household each day before and during Covid-19?

Click as many options as are relevant!

Before Covid-19

- [ ] Breakfast
- [ ] Midday meal
- [ ] Evening meal
- [ ] Random snacks
- [ ] Other (please specify):

During Covid-19

- [ ] Breakfast
- [ ] Midday meal
- [ ] Evening meal
- [ ] Random snacks
- [ ] Other (please specify):

7. What are the main ways your household food is prepared before and during Covid-19?

Click as many options as are relevant!

Before Covid-19

- [ ] Meals from the take away shop
- [ ] Take away meals from supermarket
- [ ] Ready to heat/cook meals
- [ ] Home-made mainly from processed ingredients
- [ ] Home-made mainly from raw ingredients
- [ ] Other (please specify):

During Covid-19

- [ ] Meals from the take away shop
- [ ] Take away meals from supermarket
- [ ] Ready to heat/cook meals
- [ ] Home-made mainly from processed ingredients
- [ ] Home-made mainly from raw ingredients
- [ ] Other (please specify):

8. How often do you personally eat the following food types before Covid-19?

(The During-Covid-19 options are in the next question)

Before Covid-19

Fresh vegetables and fruit

- Daily
- 4 to 6 times a week
- 2 to 3 times a week
- Once a week
- Between once a week and once a fortnight
- Less than once a fortnight or never

Fresh meat

- Daily
- 4 to 6 times a week
- 2 to 3 times a week
- Once a week
- Between once a week and once a fortnight
- Less than once a fortnight or never

Fresh fish

- Daily
- 4 to 6 times a week
- 2 to 3 times a week
- Once a week
- Between once a week and once a fortnight
- Less than once a fortnight or never

Bread

- Daily
- 4 to 6 times a week
- 2 to 3 times a week
- Once a week
- Between once a week and once a fortnight
- Less than once a fortnight or never

Dairy products

Daily
4 to 6 times a week
2 to 3 times a week
Once a week
Between once a week and once a fortnight
Less than once a fortnight or never

Frozen food

Daily
4 to 6 times a week
2 to 3 times a week
Once a week
Between once a week and once a fortnight
Less than once a fortnight or never

Canned food

Daily
4 to 6 times a week
2 to 3 times a week
Once a week
Between once a week and once a fortnight
Less than once a fortnight or never

Ready-made meals

Daily
4 to 6 times a week
2 to 3 times a week
Once a week
Between once a week and once a fortnight
Less than once a fortnight or never

Cake and biscuits

Daily
4 to 6 times a week
2 to 3 times a week
Once a week
Between once a week and once a fortnight
Less than once a fortnight or never

Chocolate, candies

Daily
4 to 6 times a week
2 to 3 times a week
Once a week
Between once a week and once a fortnight
Less than once a fortnight or never

Wine, beer and other alcoholic drinks

Daily
4 to 6 times a week
2 to 3 times a week
Once a week
Between once a week and once a fortnight
Less than once a fortnight or never

Click “Once a week” to confirm you are not a robot

Daily
4 to 6 times a week
2 to 3 times a week
Once a week
Between once a week and once a fortnight
Less than once a fortnight or never

Other (please specify):

Daily
4 to 6 times a week
2 to 3 times a week
Once a week
Between once a week and once a fortnight
Less than once a fortnight or never

9. How often do you personally eat the following food types during Covid-19?

During Covid-19

Fresh vegetables and fruit

Daily
4 to 6 times a week
2 to 3 times a week
Once a week
Between once a week and once a fortnight
Less than once a fortnight or never

Fresh meat

Daily
4 to 6 times a week
2 to 3 times a week
Once a week
Between once a week and once a fortnight
Less than once a fortnight or never

Fresh fish

Daily
4 to 6 times a week
2 to 3 times a week
Once a week
Between once a week and once a fortnight
Less than once a fortnight or never

Bread

Daily
4 to 6 times a week
2 to 3 times a week
Once a week
Between once a week and once a fortnight
Less than once a fortnight or never

Dairy products

Daily
4 to 6 times a week
2 to 3 times a week
Once a week
Between once a week and once a fortnight
Less than once a fortnight or never

Frozen food

Daily
4 to 6 times a week
2 to 3 times a week
Once a week
Between once a week and once a fortnight
Less than once a fortnight or never

Canned food

Daily
4 to 6 times a week
2 to 3 times a week
Once a week
Between once a week and once a fortnight
Less than once a fortnight or never

Ready-made meals

Daily
4 to 6 times a week
2 to 3 times a week
Once a week
Between once a week and once a fortnight
Less than once a fortnight or never

Cake and biscuits

Daily
4 to 6 times a week
2 to 3 times a week
Once a week
Between once a week and once a fortnight
Less than once a fortnight or never

Chocolate, candies

Daily
4 to 6 times a week
2 to 3 times a week
Once a week
Between once a week and once a fortnight
Less than once a fortnight or never

Wine, beer and other alcoholic drinks

Daily
4 to 6 times a week
2 to 3 times a week
Once a week
Between once a week and once a fortnight
Less than once a fortnight or never

Other (please specify):

Daily
4 to 6 times a week
2 to 3 times a week
Once a week
Between once a week and once a fortnight
Less than once a fortnight or never

10. When eating away from home, how often do you personally use the following places before and during Covid-19?

Before Covid-19

Work canteens

Daily
4 to 6 times a week
2 to 3 times a week
Once a week
Between once a week and once a fortnight
Less than once a fortnight or never

Restaurants, Cafés, Hotels or similar

Daily
4 to 6 times a week
2 to 3 times a week
Once a week
Between once a week and once a fortnight
Less than once a fortnight or never

Street vendors

Daily
4 to 6 times a week
2 to 3 times a week
Once a week
Between once a week and once a fortnight
Less than once a fortnight or never

Free food in hostels or similar

Daily
4 to 6 times a week
2 to 3 times a week
Once a week
Between once a week and once a fortnight
Less than once a fortnight or never

Other (please specify!):

Daily
4 to 6 times a week
2 to 3 times a week
Once a week
Between once a week and once a fortnight
Less than once a fortnight or never

During Covid-19

Work canteens

Daily
4 to 6 times a week
2 to 3 times a week
Once a week
Between once a week and once a fortnight
Less than once a fortnight or never

Restaurants, Cafés, Hotels or similar

Daily
4 to 6 times a week
2 to 3 times a week
Once a week
Between once a week and once a fortnight
Less than once a fortnight or never

Street vendors

Daily
4 to 6 times a week
2 to 3 times a week
Once a week
Between once a week and once a fortnight
Less than once a fortnight or never

Free food in hostels or similar

Daily
4 to 6 times a week
2 to 3 times a week
Once a week
Between once a week and once a fortnight
Less than once a fortnight or never

Other (please specify!):

Daily
4 to 6 times a week
2 to 3 times a week
Once a week
Between once a week and once a fortnight
Less than once a fortnight or never

In the questions on the following pages, we are interested in your household's behaviour and attitudes to food during the COVID-19 pandemic in comparison with before it started.

11. How much has your household´s food behaviour changed during the Covid-19 pandemic compared with before?

How much food is eaten

- Much more
- A little more
- No Change
- A little less
- Much less

How much money is spent on food

- Much more
- A little more
- No Change
- A little less
- Much less

How much unpackaged food is obtained (e.g. fruit and vegetables, bread, other bulk food)

- Much more
- A little more
- No Change
- A little less
- Much less

How much food is obtained from local producers

- Much more
- A little more
- No Change
- A little less
- Much less

How much organic food is obtained

- Much more
- A little more
- No Change
- A little less
- Much less

How much food is thrown away

- Much more
- A little more
- No Change
- A little less
- Much less

How varied is the range of food eaten

- Much more
- A little more
- No Change
- A little less
- Much less

The extent to which you plan meals and/or your grocery list in advance

- Much more
- A little more
- No Change
- A little less
- Much less

How often new recipes and/or ingredients are used

- Much more
- A little more
- No Change
- A little less
- Much less

The overall importance of food in your daily life

Much more
A little more
No Change
A little less
Much less

How far do you travel to food shops

Much more
A little more
No Change
A little less
Much less

How much alcohol is consumed

Much more
A little more
No Change
A little less
Much less

12. Has anyone in your household missed a meal because there is not enough food in the house before and during Covid-19?

Before Covid-19

- ◯ Frequently
- ◯ Occasionally
- ◯ Never

During Covid-19

- ◯ Frequently
- ◯ Occasionally
- ◯ Never

13. Has anyone in your household been anxious about obtaining enough food to meet their requirements before and during Covid-19?

Before Covid-19

- ◯ Frequently
- ◯ Occasionally
- ◯ Never

During Covid-19

- ◯ Frequently
- ◯ Occasionally
- ◯ Never

14. Has your household income changed as a result of Covid-19?

◯ Yes
◯ No

15. Does anyone in your household have any special dietary needs?

◯ Yes
◯ No

16. Does your household stock-up on food more than in the period before Covid-19?

◯ Yes
◯ No

17. Have person(s) in your household responsible for obtaining or preparing food in your household changed during the Covid-19 pandemic?

◯ Yes
◯ No

18. Is any member of your household active in, or in any way closely related to, the production, processing, distribution or delivery of food to other people apart from your own household members?

◯ Yes
◯ No

19. Have there been any other changes in your household's behaviour related to food before vs. during the Covid-19 pandemic?

◯ Yes
◯ No

20. Have there been any other changes in your household´s attitudes to food (including any lifestyle changes) before as compared to during the Covid-19 pandemic?

◯ Yes
◯ No

Please explain how your income has changed:

Please explain which special dietary needs:

What types of food do you stock more:

Please explain the changes of the specific person(s) in responsibility for obtaining or preparing food:

Please explain in which way member(s) of your houlsehold are related to production, processing, distribution or delivery of food:

Please explain those other changes due to Covid-19:

Please explain the attitude changes due to Covid-19:

21. Do you expect that any of the changes in your household's behaviour and attitudes to food during Covid-19 will continue after other aspects of your household's daily life have returned to what they were before Covid-19? - Part I

Please indicate whether any of the changes in the following areas will continue:

Types of shops used

- Definitely yes
- Probably yes
- Don´t know
- Probably no
- Definitely no

There was no change

Purchasing frequency

- Definitely yes
- Probably yes
- Don´t know
- Probably no
- Definitely no

There was no change

The money spent on food

- Definitely yes
- Probably yes
- Don´t know
- Probably no
- Definitely no

There was no change

Behaviour concerning growing own food

- Definitely yes
- Probably yes
- Don´t know
- Probably no
- Definitely no

There was no change

Other means of obtaining food

- Definitely yes
- Probably yes
- Don´t know
- Probably no
- Definitely no

There was no change

Types of food and dishes

- Definitely yes
- Probably yes
- Don´t know
- Probably no
- Definitely no

There was no change

Behaviour concerning food waste

- Definitely yes
- Probably yes
- Don´t know
- Probably no
- Definitely no

There was no change

Click “Probably no” in this line to confirm you are not a robot

- Definitely yes
- Probably yes
- Don´t know
- Probably no
- Definitely no

There was no change

22. Do you expect that any of the changes in your household’s behaviour and attitudes to food during Covid-19 will continue after other aspects of your household’s daily life have returned to what they were before Covid-19? - Part II

Please indicate whether any of the changes in the following areas will continue.

Alcohol consumption

- Definitely yes
- Probably yes
- Don´t know
- Probably no
- Definitely no

There was no change

Preparing food

- Definitely yes
- Probably yes
- Don´t know
- Probably no
- Definitely no

There was no change

Where food is eaten

- Definitely yes
- Probably yes
- Don´t know
- Probably no
- Definitely no

There was no change

The person(s) in your household responsible for food

- Definitely yes
- Probably yes
- Don´t know
- Probably no
- Definitely no

There was no change

Travel distance to food shops

- Definitely yes
- Probably yes
- Don´t know
- Probably no
- Definitely no

There was no change

Obtaining food from local producers

- Definitely yes
- Probably yes
- Don´t know
- Probably no
- Definitely no

There was no change

Overall attitudes towards food

- Definitely yes
- Probably yes
- Don´t know
- Probably no
- Definitely no

There was no change

Other (please specify):

Definitely yes
Probably yes
Don´t know
Probably no
Definitely no

There was no change

---

23. Which changes have there been in local and national regulations, commercial or other changes resulting from the Covid-19 pandemic <u>beyond your control?</u>

	Yes	No	Don´t know
Travel and movement restrictions	○	○	○
Closure or restrictions on public transport	○	○	○
Closure of restaurants, cafés, canteens	○	○	○
Closure of your (physical) workplace	○	○	○
Partial or full closure of school, educational institutions, day-care institutions	○	○	○
Closure of other public places	○	○	○
Restrictions on numbers of people in one place	○	○	○
Other (please specify):	○	○	○

If yes, please indicate the impact of these changes on your household´s behaviour and attitudes to food below.

24. Do any members of your household have Covid-19 or symptoms consistent with Covid-19 (or have they had these)?

◯ Yes
◯ No

25. Are any members of your household in isolation or in quarantine because of Covid-19 (or have they been)?

◯ Yes
◯ No

26. Are any members of your household in hospital because of Covid-19? (or have they been)?

◯ Yes
◯ No

27. What is your own perception of the risks of Covid-19?

The likelihood of any member of your household to become infected by the virus.

- Very high
- High
- Medium
- Low
- Very low

The likely severity of the virus for any member of your household (please refer to the person with the highest likely severity).

- Very high
- High
- Medium
- Low
- Very low

The level of your anxiety concerning the potential impact of the virus on your household.

- Very high
- High
- Medium
- Low
- Very low

Other (please specify): ____

- Very high
- High
- Medium
- Low
- Very low

Seite 29

dt10

To end this survey, we would like to know some facts about you. (Note: only you as respondent)

Seite 30

soc

28. Your Gender

[Please choose]

29. Your age in years:

[Please choose]

30. What is the highest educational qualification you have personally obtained?

[Please choose]

31. How many people in the following age groups live in your household (that your previous answers about food refer to), including yourself:

0-4 Years

5-9 Years

10-19 Years

20-29 Years

30-39 Years

40-49 Years

50-59 Years

60-69 Years

70-79 Years

Over 79 Years

32. Please indicate your postcode:

Seite 31

country

Seite 32

33. Please choose your country here, if you are coming from Europe:

[please choose]

34. Please choose your country here, if you are coming from outside Europe:

[please choose]

Seite 33

gt1

Seite 34

bl

Thank you for completing this questionnaire!

We would like to thank you very much for helping us.

Please add your email address here if you wish to receive more information about this study:

submit

Your answers were transmitted, you may close the browser window or tab now.
